# Targeting Hydrogel for Intelligent Recognition and Spatiotemporal Control in Cell‐Based Therapeutics

**DOI:** 10.1002/advs.202404172

**Published:** 2024-06-14

**Authors:** Weilin Hou, Wei Mao, Jun Sun, Zhiqiang Liu, Wei Shen, Hian Kee Lee, Sheng Tang

**Affiliations:** ^1^ School of Environmental and Chemical Engineering Jiangsu University of Science and Technology Zhenjiang Jiangsu 212003 P. R. China; ^2^ Central‐Southern Safety and Environmental Technology Institute Co. Ltd. Wuhan 430071 P. R. China; ^3^ School of Chemistry The University of New South Wales Sydney NSW 2052 Australia; ^4^ Department of Chemistry National University of Singapore 3 Science Drive 3 Singapore 117543 Singapore

**Keywords:** hydrogel, immune checkpoint blockade, immunogenic cell death, multimodal therapy, treatment monitoring

## Abstract

Smart drug platforms based on spatiotemporally controlled release and integration of tumor imaging are expected to overcome the inefficiency and uncertainty of traditional theranostic modes. In this study, a composite consisting of a thermosensitive hydrogel (polyvinyl alcohol‐carboxylic acid hydrogel (PCF)) and a multifunctional nanoparticle (Fe_3_O_4_@Au/Mn(Zn)‐4‐carboxyphenyl porphyrin/polydopamine (FAM^x^P)) is developed to combine tumor immunogenic cell death (ICD)/immune checkpoint blockade (ICB) therapy under the guidance of magnetic resonance imaging (MRI) and fluorescence imaging (FI). It can not only further recognize the target cells through the folate receptor of tumor cells, but also produce thermal dissolution after exposure to near‐infrared light to slowly release FAM^x^P in situ, thereby prolonging the treatment time and avoiding tumor recurrence. As FAM^x^P entered the tumor cells, it released FAM^x^ in a pH‐dependent manner. Chemodynamic, photothermal and photodynamic therapy can cause significant ICD in cancer cells. ICB can thus be further enhanced by injecting anti‐programmed cell death ligand 1, improving the effectiveness of tumor treatment. The developed PCF‐FAM^x^P composite hydrogel may represent an updated drug design approach with simple compositions for cooperative MRI/FI‐guided targeted therapeutic pathways for tumors.

## Graphical abstract

A composite consisting of a thermosensitive hydrogel and a multifunctional nanoparticle (PCF‐FAM^x^P) is developed as a drug delivery platform tailored for intelligent recognition, spatiotemporal control, and real‐time monitoring of drug release in cell‐based therapeutics.

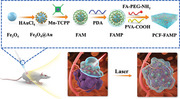

## 1 Introduction

In contemporary medical practice, immune checkpoint blockade (ICB) has emerged as the foremost approach for cancer treatment.^[^ ^1^ ^]^ This innovative therapeutic approach involves utilizing medications to disrupt the interaction between programmed cell death 1 (PD‐1) and its ligand (PD‐L1), thereby unleashing the immune system's ability to target and eliminate cancer cells.^[^ ^2^ ^]^ However, despite the remarkable success of ICB in some cases, its efficacy is limited by the poor immune response exhibited by certain patients. These individuals often present low PD‐L1 expression and insufficient *T*‐cell infiltration within the tumor microenvironment, resulting in suboptimal clinical outcomes with ICB monotherapy.^[^ ^3^ ^]^ Immunogenic cell death (ICD) of tumor cells can release tumor‐specific antigens and damage‐associated molecular patterns (DAMPs),^[^ ^4^ ^]^ triggering further activation, proliferation, and infiltration of *T*‐cells in the tumor microenvironment, and enhancing *T*‐cell activity.^[^ ^5^ ^]^ Therefore, the combination of ICD and ICB has emerged as a promising synergistic treatment approach.

In recent years, researchers have been actively developing nano enzymes to harness their potential in triggering ICD behavior.^[^ ^6^ ^]^ Examples include metal tungsten oxide materials, gold nanoparticles, platinum nanoparticles, and others.^[^ ^7^, ^8^, ^9^ ^]^ The utilization of nano enzymes presents a myriad of advantages over conventional treatment modalities, offering improved stability and versatility for combination therapy approaches.^[^ ^10^ ^]^ Despite these promising attributes, the utilization of nanomedicines is still facing intrinsic challenges. As nanomedicines exist as free molecules within the body, any deviation in their release from the target site may compromise the attainment of therapeutic concentrations, thereby diminishing their overall efficacy.^[^ ^11^ ^]^ Therefore, it is both a practical necessity and a scientific imperative to develop methods capable of precisely delivering drugs to target sites and monitoring drug distribution in real‐time, aiming to maximize the efficacy of the treatment.

The utilization of hydrogels in cancer treatment has garnered significant interest due to their distinctive properties, including excellent biocompatibility and ease of functionalization.^[^ ^12^ ^]^ This inherent biocompatibility not only mitigates the risk of adverse reactions but also renders hydrogels highly suitable for use in biological systems and as effective vehicles for drug delivery.^[^ ^13^ ^]^ Furthermore, through functionalization, hydrogels can be tailored to exhibit precise tumor‐targeting capabilities, facilitate real‐time monitoring, and enable the encapsulation of multiple therapeutic agents within a single matrix, thereby facilitating combination therapies.^[^ ^14^ ^]^ Despite these advantages, the majority of research efforts in the field of hydrogel drug delivery systems have been predominantly focused on enhancing cancer treatment efficacy or improving tumor visualization.^[^ ^15^ ^]^ However, the integration of targeted therapy with advanced imaging techniques remains relatively underexplored. Nevertheless, this approach holds great promise for optimizing the treatment process and expediting clinical decision‐making for patients. To fully unlock the potential of hydrogel‐based drug delivery systems in oncology, research should aim to bridge the gap between advanced treatment, targeted therapy, and imaging modalities.^[^ ^16^ ^]^

**Scheme 1:**
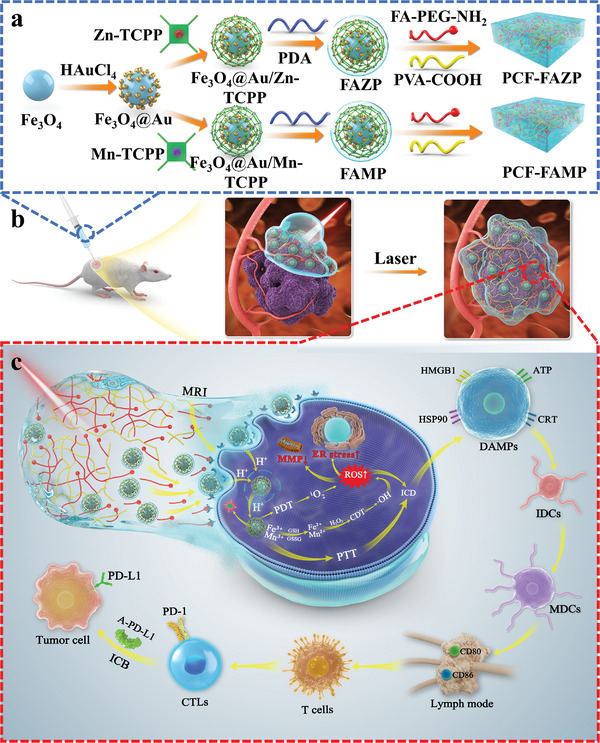
a) Synthetic scheme of PCF‐FAM^x^P; b) schematic for injecting PCF‐FAM^x^P for encapsulation of tumors; c) schematic of the specific recognition of PCF‐FAM^x^P in tumor cells to restore fluorescence and synergistically trigger the ICD behavior of tumor cells through chemodynamic therapy (CDT), photodynamic therapy (PDT) and photothermal therapy (PTT).

In this study, we developed a multifunctional hydrogel‐based drug delivery system tailored for intelligent recognition, spatiotemporal control, and real‐time monitoring of drug release in cell‐based therapeutics. The core material, Fe_3_O_4_@Au, was coupled with Mn‐ or Zn‐4‐carboxyphenyl porphyrin (TCPP) and encased within a polydopamine (PDA) membrane, yielding two distinct composites: Fe_3_O_4_@Au/Zn‐TCPP/PDA (FAZP) and Fe_3_O_4_@Au/Mn‐TCPP/PDA (FAMP). These two composite materials were then incorporated into a folic acid modified polyvinyl alcohol‐carboxylic acid (PVA‐COOH) hydrogel matrix (PCF), resulting in the construction of the PCF‐PAZP/PCF‐FAMP (PCF‐FAM^x^P) drug delivery platform (**Scheme** **1**). Compared to conventional anti‐cancer drug delivery systems, the platform possessed several notable advantages: 1) A multi‐pathway therapeutic approach involving ICD and ICB through the utilization of nanomaterials; 2) long‐distance stable drug delivery and spatiotemporal control of drug release via the PCF‐PAM^x^P functionalized hydrogel platform; and 3) real‐time monitoring of drug delivery. The platform enabled real‐time observation of drug delivery and tumor treatment efficacy through fluorescence imaging (utilizing metal‐TCPP) and magnetic resonance imaging (MRI)‐assisted external monitoring (utilizing Fe_3_O_4_), providing potentially valuable information into treatment progress and response.

## 2 Results and Discussion

### 2.1 Synthesis and Characterization of Materials

**Figure 1:**
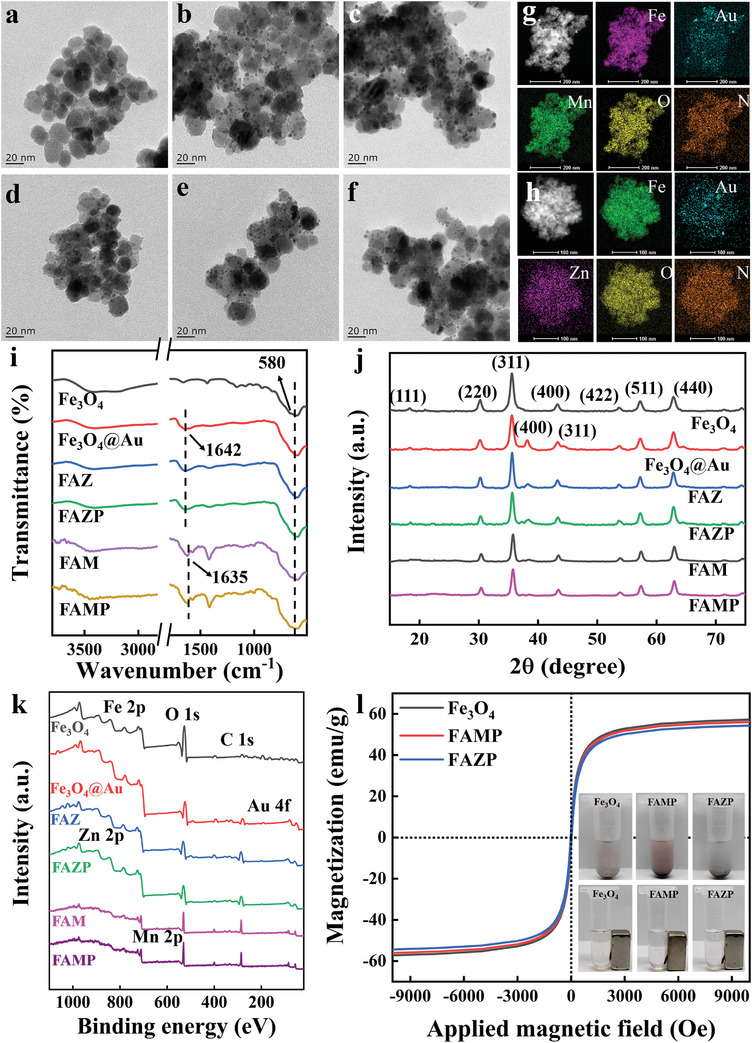
TEM images of a) Fe_3_O_4_; b) Fe_3_O_4_@Au; c) FAM; d) FAZ; e) FAMP; f) FAZP; g) TEM images of FAMP and the insets show corresponding elemental mappings of the Fe, Au, Mn, O and N; h) TEM images of FAZP and the insets show corresponding elemental mappings of the Fe, Au, Zn, O and N; i) FT‐IR spectra; j) XRD spectra; k) XPS spectra; l) magnetization curves.

The Fe_3_O_4_ was synthesized through the precipitation method. As illustrated in **Figure** **1**a, the size of the Fe_3_O_4_ obtained was ca. 20–30 nm. The Au was formed in situ on the surface of Fe_3_O_4_ through the reduction of the chloroauric acid anion by the addition of sodium borohydride and sodium citrate; the particle size of Au was about 3 nm (Figure 1b). After the addition of Mn‐TCPP or Zn‐TCPP on the Fe_3_O_4_@Au surface (Figure 1c,d), there was no visible change in terms of the structure of Fe_3_O_4_@Au. To establish a stable protective environment for Fe_3_O_4_@Au/Mn(Zn)‐TCPP (FAM^x^), minimizing the risk of transportation‐related losses and potential hazards, a PDA coating was attached to the FAM^x^ surface (Figure 1e,f). High‐resolution transmission electron microscopy (HRTEM) images and elemental map analysis (Figure 1g,h) were performed to further explore the synthesized materials. These analyses revealed a uniform distribution of all elements, including Fe, Au, Zn/Mn, O, and N, further confirming the successful synthesis of FAM^x^P. According to the Fourier transform infrared (FT‐IR) spectra of the obtained materials (Figure 1i), the strong absorption band at 580 cm^−1^ was caused by Fe–O vibrations in Fe_3_O_4_.^[^ ^17^ ^]^ The band 1642 cm^−1^ corresponded to the N─H bond resonance of polyethyleneimine (PEI),^[^ ^18^ ^]^ so it demonstrated that the surface of Fe_3_O_4_ had been positively modified by PEI. The peak appearing at 1635 cm^−1^ can be attributed to the aromatic ring skeleton stretching vibration, confirming the PDA coating on the surface.^[^ ^19^ ^]^ Fe_3_O_4_ remained stable as the core in later synthesis. X‐ray diffractometry (XRD) was used to explore the structure during the synthesis of FAMP (Figure 1j). In the diffraction peaks of Fe_3_O_4_, the peaks at 2θ of 18.3°, 30.6°, 35.8°, 43.4°, 53.6°, 57.5°, and 63.2° corresponded to the (220), (311), (400), (422), (511) and (440) crystal planes of Fe_3_O_4_, respectively, demonstrating the successfully synthesis of Fe_3_O_4_. Similarly in the characterization of Fe_3_O_4_@Au, the diffraction peaks 2 θ of 38.2° and 44.4° corresponded to the (400) and (331) crystal planes of Au.^[^ ^17^ ^]^ After the M^x^‐TCPP modification and the PDA wrapping, the characteristic peaks of Fe_3_O_4_ and Au remained without much change, showing that in the FAM^x^, the structures of Fe_3_O_4_ and Au were maintained. Additionally, the Fe_3_O_4_‐based nanomaterials displayed good magnetic properties (Figure 1l). As illustrated in Figure 1l, all Fe_3_O_4_‐based nanomaterials can be quickly separated from aqueous solutions by using an external magnet. The elemental composition of each of the Fe_3_O_4_, Fe_3_O_4_@Au, FAM^x^ materials was further evaluated by X‐ray photoelectron spectroscopy (XPS) (Figure 1k). XPS images showed the presence of Zn and Mn elements in FAZ and FAM; the stability of M^x^‐TCPP during synthesis was also demonstrated, laying the groundwork for successful fluorescence imaging. The Fe 2p spectrum had distinct Fe^2+^ (711.8 and 725.3 eV) and Fe^3+^ (715.4 and 729.4 eV) binding energy peaks, indicating the presence of Fe_3_O_4_ (Figure S1a, Supporting Information). The characteristic peaks of Au^3+^ (84.6 and 88.4 eV) and Au^0^ (84.1 and 87.8 eV) indicated the presence of elemental Au (Figure S1b, Supporting Information).^[^ ^20^ ^]^

### 2.2 Synthesis of PCF‐FAM^x^P

**Figure 2:**
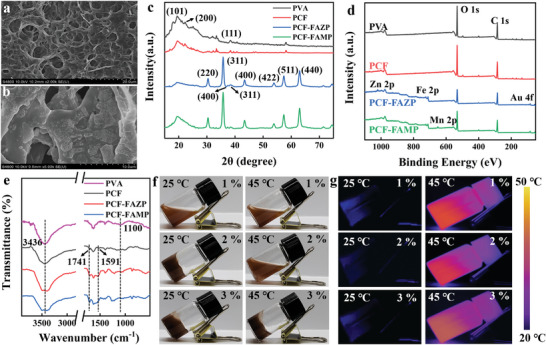
SEM images of a) PCF; b) PCF‐FAMP; c) FT‐IR spectra; d) XPS spectra; e) XRD spectra; f,g) thermographic images of hydrogel with PVA‐FAMP concentrations of 1%, 2%, and 3% at 25 and 45 °C.

The PCF hydrogel was used as the smart drug platform. The internal porous structure can be clearly observed in **Figure** **2**a. Scanning electron microscopic (SEM) images confirm the successful loading of nanomedicine onto the PCF hydrogel (Figure 2b). XPS and XRD were used to further demonstrate successful PCF‐FAMP synthesis. Sharper crystallographic diffraction peaks at 20.12°, 23.42°, and 41.18°, correspond to the PVA (101) mixed crystalline diffraction peak, (200) crystalline diffraction peak, and (111) crystalline diffraction peak, respectively (Figure 2c).^[^ ^21^ ^]^ The introduction of PCF hydrogel did not lead to changes in the characteristic peaks. Also the XPS analysis presented its characteristic elements (Figure 2d), demonstrating the successful drug loading on the hydrogels. The 1100 cm^−1^ peak corresponded to the C–O stretching vibration of PVA; the broad band at 3436 cm^−1^ was due to the O–H stretching of inter‐ or intramolecular hydrogen bonds present in PVA.^[^ ^22^ ^]^ A strong band at 1741 cm^−1^ was seen in its IR characterization to confirm the C═O stretching of the ─COOH group.^[^ ^23^ ^]^ The characteristic amide N–H peak at 1591 cm^−1^ showed the aminocarboxylic binding of carboxylated hydrogels to folic acid.^[^ ^24^ ^]^ The enhancement of the characteristic peaks of binding with nanodrugs also proved that the drug and hydrogel can be bound directly and also through organic functional groups, greatly improving its load factor (Figure 2e). To ensure the good mechanical stability of the PCF hydrogel the optimization of PCF concentration was conducted. FAMP doped with different concentrations of the PCF hydrogel can be observed from the bright‐field images and thermographic images at 25 °C and 45 °C through 808 nm laser irradiation (Figures 2f,g). At a PCF‐FAMP concentration of 1%, the hydrogel exhibited insufficient mechanical strength, leading to instability at both 25 °C and 45 °C. However, with a PCF‐FAMP concentration of 2%, the hydrogel demonstrated stability at 25 °C and transitioned to a sol state at 45 °C, aligning with our experimental criteria for injectable gels. Upon increasing the PCF‐FAMP concentration to 3%, the hydrogels displayed an undesirable excess of mechanical strength, retaining their original shape even at 45 °C. This property is not conducive for injection. To further explore the temperature‐induced gel–sol phase transition behavior, frequency sweeps ranging from 0.1 to 100 Hz and temperature sweeps from 25 °C to 80 °C were conducted at a constant strain of 1%, to assess the stability of the PCF‐FAMP hydrogel. The oscillation frequency testing results showed that PCF‐FAMP hydrogels under different temperatures had different features under low/high frequency, respectively. At 25 °C and 45 °C, respectively, the energy storage modulus (G') and loss modulus (G″″) of the 1% PCF‐FAMP hydrogels did not differ significantly (Figure S2a, Supporting Information) and were in a sol‐gel state. Two percent PCF‐FAMP hydrogels showed G' > G″″ at 25 °C, which represented the gel state. When the temperature reached 45 °C, G' < G″″, the transition was to a sol‐gel state. Thus, the 2% PCF‐FAMP hydrogel was characterized by a gel–sol transition at that specified temperature (Figure S2b, Supporting Information). However, the 3% PCF‐FAMP hydrogel had strong mechanical properties and remained in the gel state at 45 °C, which could not lead to good experimental results (Figure S2c, Supporting Information). Meanwhile, the rheological tests provided data on G' and G″″ of the hydrogel encapsulating the material at various temperatures. At 25 °C versus 45°C, when the PCF‐FAMP concentration was 1%, G' and G″″ were nearly equal, indicating a preference for the sol state structure at this temperature (Figure S3a, Supporting Information). When the concentration was 2%, at 25 °C versus 45 °C, and with increasing temperature, the rise in G' was less pronounced compared to the rise in G″″, bringing G″″ closer to G'. Consequently, the hydrogel gradually favored the gel–sol state (Figure S3b, Supporting Information). At 25 and 45 °C, for a 3% PCF‐FAMP concentration, and with increasing temperature, G' exhibited a more significant increase than G″″ (Figure S3c, Supporting Information). In this case, the hydrogel maintained its gel state. Concurrently, the findings from the frequency scan aligned with those of the temperature scan, demonstrating that a 2% hydrogel underwent a gel‐to‐sol transformation. The results from the extrusion experiments were successfully validated (Figures S4, Supporting Information), and indicated that PCF‐FAMP hydrogel was amenable to being syringed. This indicates that our hydrogel could wrap around the tumor, establishing favorable conditions for tumor treatment.

### 2.3 Exploration of CDT/PDT/PTT Activity In Vitro

**Figure 3:**
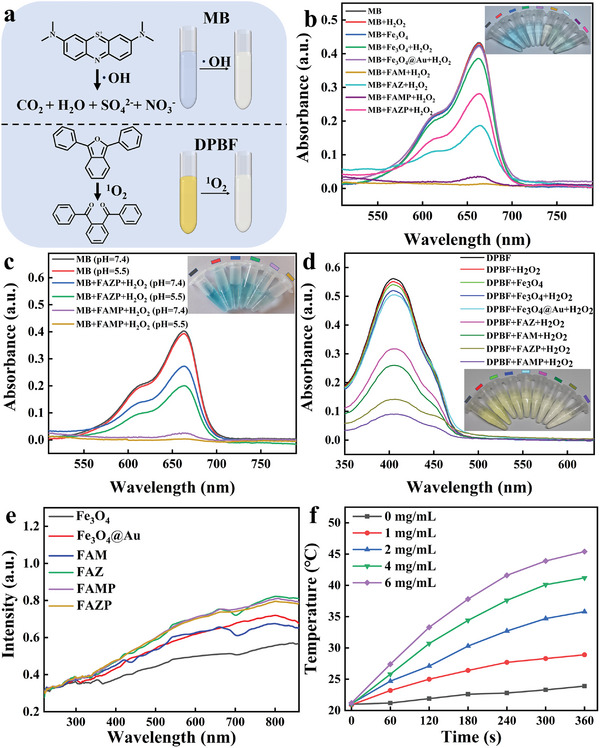
a) Schematic of chromogenic principle of MB and DPBF; b) comparison of Fenton reaction effects; c) Fenton reaction effects of FAMP and FAZP at pH = 7.4 and pH = 5.5; d) comparison of DPBF effects; e) UV–visible spectroscopic spectra of materials; f) temperature changes of FAMP aqueous suspensions with different concentrations under laser (808 nm, 1.0 W cm^−2^). Data are represented as mean ± SD (*n* = 3).

Due to the inclusion of Fe^3+^, Fe_3_O_4_ demonstrates noteworthy Fenton catalytic activity,^[^ ^25^ ^]^ which can conceivably bring exceptional CDT performance to FAM. To assess the hydroxyl radicals (·OH) generation capability, methylene blue (MB) was employed as an indicator for analysis. The ·OH produced by the material leads to the degradation of MB, resulting in a decrease of the absorption peak in the MB solution at 665 nm (**Figure** **3**a).^[^ ^26^ ^]^ It is evident that under neutral conditions, neither hydrogen peroxide or Fe_3_O_4_ alone yielded ·OH, resulting in minimal observable changes in MB (Figure 3b). Nonetheless, the introduction of Mn‐TCPP in FAM demonstrated a heightened generative capacity in comparison to Fe_3_O_4_@Au. The incorporation of Mn‐TCPP as a dopant is particularly noteworthy, as the isolated Mn^3+^ served to augment the CDT properties of the nanomedicine.^[^ ^27^ ^]^ Furthermore, the degradation of MB observed with FAMP was less effective compared to that of the material without the protective PDA film. This phenomenon underscores the validation that PDA films serve as a protective barrier for internal (core) materials, particularly under neutral conditions. In general, blood maintains a neutral pH in terms of human intracellular environment. In contrast, the tumor has an acidic microenvironment with an approximate pH value of 6.8; the intracellular environment (of tumor cells) is also acidic (pH of ca. 4.5–5.5).^[^ ^28^ ^]^ To further evaluate the selectivity of FAMP for the tumor cells, the degradation of MB by using FAMP under both pH = 7.4 and pH = 5.5 was evaluated. Notably, a considerable improvement in degradation was evident under acidic conditions (Figure 3c). This observation implies that PDA membranes experience cleavage in acidic environments, revealing the internal materials and promoting subsequent reactions. To verify the exceptional reactive oxygen species (ROS) properties of our synthesized nanomedicines, 1,3‐biphenyl isocoumarin‐2,5‐diphenyl‐3,4‐benzofuran (DPBF) was used as an indicator to monitor the generation of singlet oxygen (^1^O_2_). Upon oxidation of DPBF by ^1^O_2_, a distinct color change from yellow to colorless was observed (Figure 3a), accompanied by a reduction in the absorption peak at ca. 418 nm.^[^ ^29^ ^]^ In comparison with a blank control, the absorption peaks of FAMP exhibited a noticeable decline (Figure 3d). These results strongly indicated the capacity of our nanomedicine to execute ROS stress strategies. Meanwhile, Au can generate localized plasmon resonance upon exposure to light, leading to robust light absorption within a specific range of wavelengths. The absorbed energy can subsequently be converted into thermal energy^[^ ^30^ ^]^ for facilitating drug release from the hydrogel, as well as promoting PDT and PTT processes within the tumor. The absorbed light range of each material was assessed through UV–visible absorption spectroscopy (Figure 3e). Fe_3_O_4_ showed no prominent absorption peaks, whereas other materials, owing to the presence of Au, demonstrated optimal light absorption at wavelengths around 800 nm.^[^ ^31^ ^]^ To assess the photothermal efficiency of FAM as PDT and PTT agents, a laser with a wavelength of 808 nm was employed for irradiation and temperature changes were monitored using an IR thermographic camera. Upon laser irradiation at 808 nm (1.0 W cm^−2^) for 5 min, the temperature of the FAMP solution at a concentration of 6 mg mL^−1^ rose to 45 °C (Figure 3f), demonstrating concentration‐dependent PTT performance (Figure S5, Supporting Information). Therefore, a FAMP solution with a concentration of 6 mg mL^−1^ was selected for subsequent experiments.

### 2.4 Fluorescence Monitoring

**Figure 4:**
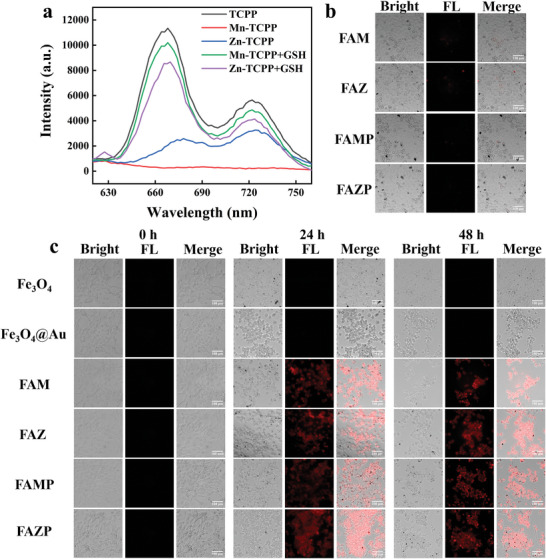
a) GSH restores the fluorescence (FL) of Mn‐TCPP and Zn‐TCPP; b) bright‐field images, FL images and merge images of FAM, FAZ, FAMP, and FAZP incubated with H8 cells for 48 h; c) bright‐field images, FL images and merge images of FAM modified with different concentrations of PDA incubated with Hela cells for 0, 24 and 48 h; d) bright‐field images, FL images and merge images of Fe_3_O_4_, Fe_3_O_4_@Au, FAM, FAZ, FAMP and FAZP incubated with HeLa cells for 0, 24 and 48 h.

As mentioned before, the microenvironment of tumor cells is more acidic compared to a normal cell. The acidic microenvironment has the potential to break down the PDA membrane of FAMP, imparting to it the capability for pH‐dependent release control under acidic conditions. The thickness of the PDA film plays a crucial role in enabling FAM to exhibit its optimal performance. To address this, we conducted optimization of the FAMP synthesis by varying the concentration of the PDA solution, as illustrated in Figure S6 (Supporting Information). At PDA concentrations of 1 and 2 mg mL^−1^, both fluorescent staining and the cytotoxic effect on human cervical cancer (HeLa) cells were minimally impacted. However, when the PDA concentration reached 3 mg mL^−1^, the growth of HeLa cells was noticeably less inhibited, and the rate of fluorescent staining slowed down. This observation suggests that PDA at higher concentrations may act to protect FAM materials, influencing their interaction with HeLa cells. In our final selection, a PVA solution concentration of 2 mg mL^−1^ was chosen for the synthesis of FAMP. To monitor the real‐time treatment of tumor cells with nanomedicines, we employed TCPP as a fluorescent imaging agent within the FAMP system. The fluorescence of TCPP underwent self‐quenching upon coordination chelation with M^x^ (Mn^3+^ and Zn^2+^).^[^ ^32^ ^]^ However, in an environment of tumor cells with high levels of glutathione (GSH).^[^ ^33^ ^]^ M^x^‐TCPP would experience competition from it, leading to the restoration of fluorescence in TCPP, as illustrated in **Figure** **4**a.^[^ ^34^ ^]^ This competitive interaction was confirmed in the cytofluorogram, where GSH competed with M^x^‐TCPP, resulting in the restoration of fluorescence to TCPP. This property makes TCPP suitable for use as a fluorescent stain for tumor cells. To verify its practical efficacy, all synthesized materials were introduced to HeLa cells, and an inverted fluorescence microscope was employed to observe the cells (Figure 4c). All the materials did not exhibit fluorescence at the start of the experiment. Following 24 h of incubation, Fe_3_O_4_ and Fe_3_O_4_@Au did not show any fluorescence as they did not contain TCPP. FAM was the first to manifest its therapeutic effects, entering the cell and inducing cytotoxicity to eliminate the cell without hindrance from the PDA membrane. In contrast, FAM gradually exerted its therapeutic effects while encapsulated within the PDA membrane. Notably, all four materials exhibited fluorescence in the presence of intracellular GSH. The incubation persisted for 48 h, and the most rapid cell elimination continued to be observed with the FAM material. Additionally, for FAMP, the efficiencies of cell elimination increased which demonstrated an increasing rate of tumor cell destruction. The visualization of cell survival was clearly evident via fluorescence throughout the process. However, operating within the organism's environment involves not only eliminating tumor cells but also striving to avoid harming healthy cells. To address this, comparative tests on human normal cervical epithelial cells (H8) were conducted, as depicted in Figure 4b. Due to the limited expression of hydrogen peroxide in normal cells,^[^ ^35^ ^]^ the materials FAM^x^ and FAM^x^P did not notably impact the normal growth of H8 cells. However, in the absence of PDA membrane protection, FAM still exhibited a minor extent of fluorescence induced by GSH. This underscores the importance of PDA for providing additional protection in such scenarios.

### 2.5 ICD Performance In Vitro

**Figure 5:**
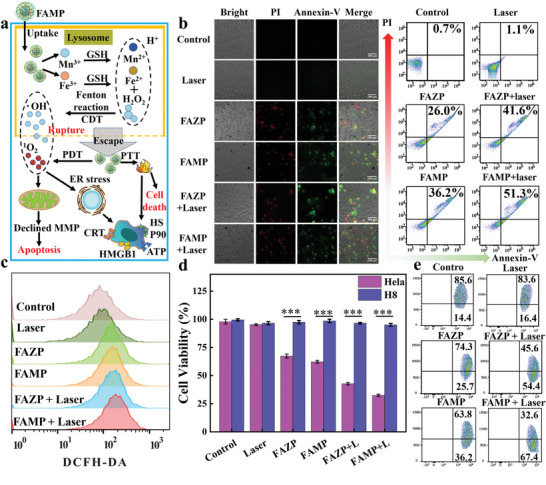
a) Schematic of FAM lysosomal escape and ICD induction by CDT/PDT/PTT; b) bright‐field images, fluorescent inverted microscope images and merge images of Control, Laser, FAZP, FAMP, FAZP + laser and FAMP + laser treated HeLa cells; c) FCM analysis of intracellular ROS levels; d) relative cell viability of cells after different treatments (*n* = 3). Student's *t*‐test, ^*^ *p* < 0.05, ^**^ *p* < 0.01, ^***^ *p* < 0.001; e) FCM analysis of MMP changes.

Lysosomes originate from the continual division and fusion of numerous vesicles under an acidic environment.^[^ ^36^ ^]^ In general, ICD is induced through the combined processes of lysosomal escape and ROS/PTT.^[^ ^2^ ^]^ As depicted in the schematic of induced ICD (**Figure** **5**a), FAMP was absorbed by cellular endocytosis. The acidic environment of the lysosome^[^ ^37^ ^]^ induced PDA membrane cleavage, with the release of Fe‐TCPP and Mn‐TCPP. Subsequently M^x^‐TCPP faced competition by GSH, with the liberated Fe^3+^/Mn^3+^ undergoing reduction to form Fe^2+^/Mn^2+^ in the presence of abundant reducing substances, primarily GSH. This reduction initiated the Fenton reaction, generating ·OH, which induced peroxidation of the lysosomal membrane. Subsequently Fe_3_O_4_@Au escaped from the lysosome into the cytoplasm and under laser irradiation at 808 nm, PTT and PDT were produced thereby inducing ICD to achieve the therapeutic effect. HeLa cells were stained with Annexin V/PI. Without laser irradiation, FAMP functioned solely in a CDT role. However, upon introducing laser conditions, a noticeable reduction in the activity of HeLa cells was observed. This phenomenon further suggested that the combined therapeutic effects of CDT, PDT, and PTT were at play. It was found that the FAMP + laser group was occupied by strongly red fluorescence and strongly green fluorescence (Figure 5b) with an apoptosis rate of 59.26%, indicating a good cell‐killing effect. Meanwhile, HeLa cells and H8 cells were subjected to cellular activity assays; the worst cell activity was observed in the FAMP + laser group (Figure 5d), which was consistent with the described results. These latter further confirmed the treatment effect of nanomedicines, laying the foundation for in vivo therapy. However, to verify whether the ICD effect was successfully induced, the biomarkers related to DAMPs were evaluated. The evaluation included the release of high mobility group box protein 1 (HMGB1), notable overexpression of calreticulin (CRT), measurement of adenosine triphosphate (ATP) secretion,^[^ ^38^ ^]^ and heat‐shock proteins 90 (HSP90) exposure.^[^ ^39^ ^]^ These parameters were tested using ELISA under identical experimental conditions. HMGB1 and ATP were applied to activate antigen‐presenting cells,^[^ ^40^ ^]^ in comparison to the control group (see Figure S7a, Supporting Information). The levels of intracellularly released HMGB1 increased by 1.61‐fold and 2.03‐fold in the FAM^x^P‐induced CDT group and the FAM^x^P + laser‐induced CDT/PDT/PTT combination treatment group, respectively. In the same groups, according to Figure S7b (Supporting Information), the intracellular ATP levels experienced 1.62‐fold and two‐fold increases, respectively. CRT is accountable for glycoprotein folding, functioning as an “eat me” signal, and it can translocate from the endoplasmic reticulum (ER) to the cell surface to aid in antigen presentation.^[^ ^41^ ^]^ The high expression of CRT was likewise observed (Figure S7c, Supporting Information), indicating the degree of apoptosis. Notably, HSP90, a member of the cellular stress protein family, has the capability to translocate from the intracellular membrane to the plasma membrane under high‐temperature stimulation or other stress conditions.^[^ ^42^ ^]^ In the FAM^x^P + laser group, HSP90 responded to oxidative stress induced by PDT/PTT. Therefore, notably higher expression was observed (see Figure S7d, SI). Considering the aforementioned properties of FAM^x^P, the in vitro performance of nanomedicines was assessed. The effectiveness of intracellular ·OH and ^1^O_2_ production by the nanoadjuvants was evaluated using a 2,7‐dichlorodihydrofluorescein diacetate (DCFH‐DA) probe. ROS (·OH and ^1^O_2_) production disrupted ER homeostasis, ultimately causing ER stress to drive the ICD effect.^[^ ^43^ ^]^

CDT and PDT can cause changes in intracellular ROS. So, the quantitative results obtained were analyzed using flow cytometry (FCM). In contrast to the control group, the FAM^x^P group showed significant changes. Meanwhile the FAMP + laser group reached the highest intracellular ROS levels with the combination of both treatments due to the stronger Fenton effect of Mn^3+^ and the PDT produced by laser irradiation (Figure 5c). ·OH production was accompanied by GSH consumption (Figure 5a). Next, the intracellular GSH levels were assessed using a GSH assay kit. Consistent with the ROS results, the decrease in GSH levels confirmed the production of CDT and PDT (Figure S8, Supporting Information). Since mitochondria are involved in the apoptotic pathway,^[^ ^44^ ^]^ and intracellular ROS may also cause mitochondrial dysfunction,^[^ ^45^ ^]^ leading to changes in mitochondrial membrane potential (MMP), JC‐1 staining was used to assess mitochondrial damage.^[^ ^46^ ^]^ MMP was dramatically decreased by treatment with FAMP + laser group (Figure 5e). These data suggested that the nanomedicine had excellent apoptotic effects and may have further anti‐tumor effects in vivo.

### 2.6 In Vivo Tumor Therapy

**Figure 6:**
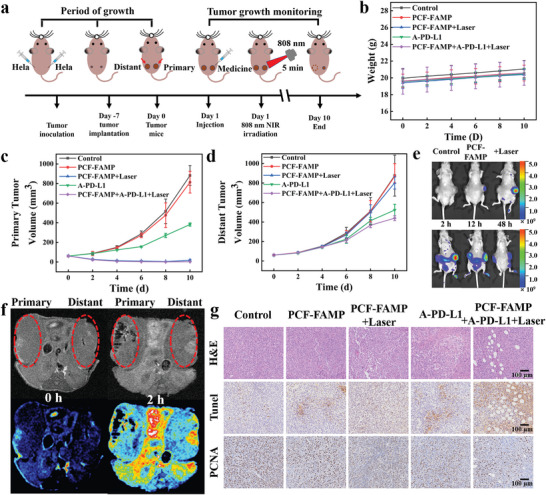
a) Schematic of the PCF‐FAMP for primary tumor on female nude mice; b) body weight records of mice after treatments; c) treated tumor volume changes of six groups after various treatments; d) distant tumor volume changes of six groups after various treatments; e) fluorescence distribution measurement of mice at 48 h; f) T 1‐weighted and T 1‐mapping MR images recorded at tumor region after intravenous injection; and g) photographs of mice and H&E/TUNEL/PCNA immunochemical staining of tumor sections after various treatments. (Scale bar: 50 µm). Data are represented as mean ± SD (*n* = 5).

Further research was conducted on the anti‐tumor effects in vivo. Following the treatment timeline, the in vivo antitumor performance was then evaluated with HeLa tumor‐bearing nude mice as models (**Figure** **6**a). The mice were randomly divided into five groups for different treatments: I) Control; II) PCF‐FAMP; III) PCF‐FAMP + laser (1.0 W cm^−2^); IV) PD‐L1 antibody (A‐PD‐L1); and V) PCF‐FAMP + laser (1.0 W cm^−2^) + A‐PD‐L1. Group (II), group (III), and group (V) were injected in situ at the tumor site with 100 µL of PCF‐FAMP drug (2% PCF hydrogel with 6 mg mL^−1^ of FAMP). Groups (III) and (V) were administered with laser irradiation for 10 min on the first and second day. In addition, group (IV) and group (V) were injected with A‐PD‐L1 (1 mg k^−1^g, in 200 µL of phosphate buffered saline (PBS) for each mouse) on day 1. The treatment ended after 10 days. Mouse body weight and tumor volumes were recorded during treatment (Figure 6b–d). The weight of tumor‐bearing mice treated with different materials display no significant changes, indicating a good biosafety profile (Figure 6b).^[^ ^47^ ^]^ As shown in Figure S9 (Supporting Information), group (III) and group (V) were irradiated with laser light for 6 min, the temperature of the tumor region rapidly increased to about 50 °C. The transition temperature of the hydrogel form (gel to sol) can be reached. In group (I), the primary tumors and distant tumors grew rapidly due to the absence of drug inhibition. Without laser irradiation after drug injection in group (II), the PCF hydrogel could not turn into a sol state and the drug could not be released immediately. Over time, the slow‐released drug was absorbed by the cells at the tumor site, and in the absence of laser irradiation, only a CDT effect could be produced. The primary tumors and distant tumors could not be effectively inhibited. In contrast, laser irradiation was performed immediately after drug injection in group (III), and the ICD effect induced by the combination of CDT, PDT and PTT highly inhibited the growth of primary tumors. However, this did not significantly change the growth of distant tumors, which prevented the achievement of the desired therapeutic effect. For group (IV) tumor‐bearing mice were used to assess the in vivo anti‐tumor effects of ICB‐based A‐PD‐L1 immunotherapy, which achieved moderate inhibition of primary tumors as well as some mild inhibition of distant tumors. For group (V) a combination of ICD and ICB treatment was applied, which highly inhibited the growth of the primary tumors, as well as some moderate inhibition of the distant tumors. This indicated that the envisioned combined treatment of ICD and ICB had good therapeutic effects on the tumors (Figures 6c,d). The images of tumors in vivo tumors after the different treatments (Figures S10a,b, Supporting Information) closely mirrored the tumor growth pattern discussed earlier, suggesting that the in vivo antitumor treatment was effective. In addition, hematoxylin and eosin (H&E), immunochemical staining with terminal deoxynucleotidyl transferase‐mediated dUTP nick‐end labeling (TUNEL) and proliferating cell nuclear antigen (PCNA) were performed to visualize the histological features of the primary tumor tissue. From the H&E staining and TUNEL results, group (V) caused more necrosis in the tumor tissue and apoptotic tumor cell counts among the groups. Moreover, PCNA assay showed almost no proliferation activity of tumor cells in group (V), suggesting that the combination treatment had proliferation inhibitory efficacy on tumor cells (Figure 6g).

Multimodal imaging holds great promise for cancer diagnosis and treatment because it allows visualization of tumor characteristics and monitoring of treatment effects using information from different imaging modalities.^[^ ^7^ ^]^ So, the in vivo distribution and treatment of PCF‐FAMP in tumor‐bearing mice were monitored using fluorescence and MR dual‐mode imaging. After in situ injection of the drug and laser irradiation, FAMP began to be released and was taken up by the cells, and drug diffusion was observed at 2 h. It is clear that after 48 h, the drug spread to both ends of the tumor, indicating the successful delivery of the drug (Figure 6e). MRI has the advantages of high resolution, nonradioactivity, and unlimited imaging depth, and so is an important diagnostic imaging modality.^[^ ^48^ ^]^ The Fe and Mn elements of FAM itself are paramagnetic and can be used as an excellent contrast agents for T1‐weighted MRI.^[^ ^49^ ^]^ Therefore, the latter was performed after in situ drug injection. Two hours after intravenous injection, T1‐weighted MRI of the tumor site exhibited increased brightness. T1 mapping further demonstrated the effective accumulation of nanoadjuvants at the tumor site (Figure 6f). The results demonstrated that the present materials had good tumor therapeutic effects and excellent tumor imaging capabilities.

### 2.7 Analysis of Antitumor Immunity Induced by PCF‐FAMP

**Figure 7:**
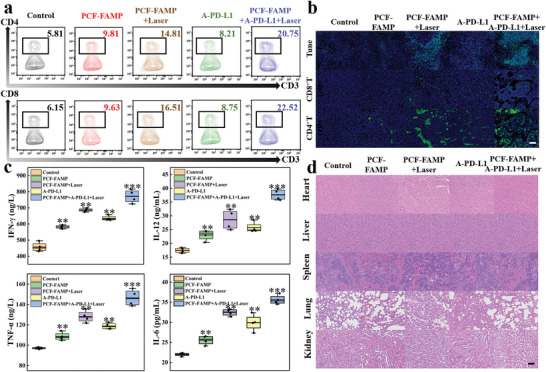
a) FCM analysis of CD4^+^/CD8^+^ *T*‐cells expression of tumors; b) immunochemical staining of TUNEL on the primary tumor and immunochemical staining of CD4^+^/CD8^+^ *T*‐cells; c) the level of IL‐6, IL‐12, TNF‐α, and IFN‐γ in serum of different groups by Elisa (*n* = 4). Student's *t*‐test, ^*^ *p* < 0.05, ^**^ *p* < 0.01, ^***^ *p* < 0.001; d) H&E staining of the major organs at corresponding time intervals.

Limited infiltration of antigen‐specific antitumor *T*‐cells in tumors hinders cancer immunotherapy.^[^ ^46^ ^]^ Therefore, the number of cytotoxic *T*‐cells (CD8^+^) and helper *T*‐cells (CD4^+^) reflects the extent to which the immune reaction against a tumor is underway.^[^ ^50^ ^]^ Considering that the immune response of distant tumors reflects the primary tumor and systemic immune activation,^[^ ^51^ ^]^ in this work the immune cellular response to the distant tumor was assessed. The numbers of intratumoral CD4^+^ *T*‐cells and CD8^+^ *T*‐cells were studied by FCM and immunofluorescence staining. Consistent with the trend of growth restriction in distant tumors, compared to the control group. Increased numbers of CD4^+^ and CD8^+^ T cells were observed in the monotherapy groups (PCF‐FAMP, PCF‐FAMP + laser, and A‐PD‐L1,). The combination treatment of ICD with ICB (PCF‐FAMP + A‐PD‐L1 + laser) induced significantly higher numbers (20.75% and 22.52%, respectively), exhibiting the highest *T*‐cell (CD4^+^ and CD8^+^) activity (**Figure** **7**a). Subsequently, immunofluorescence staining of the treated distant tumor tissue was conducted to assess the level of damage and immune response to the tumor. The trend of results from H&E staining, TUNEL, and PCNA was consistent with proximal tumors, and the combination therapy also showed some inhibitory effect on distant tumors. In comparison to the control group, the monotherapy group showed moderate fluorescence of tumor‐infiltrating T cells, indicating that ICD or ICB was successfully induced. When the two immunotherapies were integrated, intense fluorescence was observed (Figure 7b). This indicated that PCF‐FAMP + laser‐mediated ICD could work reasonably efficiently in coordination with ICB to promote proliferation of tumor infiltrating *T*‐cells, causing further suppression of the growth of tumors. In addition to immune‐related cells, cytokines play a key role in triggering an immune response.^[^ ^52^ ^]^ When the immune response is triggered, immune cells release large amounts of tumor necrosis factor (TNF)‐α and interferon (IFN)‐γ,^[^ ^53^ ^]^ which can promote apoptosis of tumor cells and modulate the function of immune cells.^[^ ^51^, ^54^ ^]^ At the same time, dying cancer cells release cytokines (interleukin (IL)−12 IL‐6) that will enhance the immune response to TME during ICD.^[^ ^55^ ^]^ As shown in Figure 7c, the expression levels of TNF‐α, IFN‐γ, (IL)−6 and IL‐12 were significantly higher in the monotherapy group, while the combination therapy group showed the highest levels of the immune markers. These results suggest that PCF‐FAMP + A‐PD‐L1 + laser can trigger an effective immune response and thus inhibit tumor growth. Major organs of mice in each treatment group were obtained for H&E staining to further assess the biosafety of PCF‐FAMP. At the test dose, no significant histopathologic changes were observed in major organs (Figure 7d), indicating no toxicity to mice. This further demonstrated the high potential clinical application of the developed nanoadjuvant.

## 3 Conclusion

In this study, we synthesized FAMP nanomaterials by layer stacking and combined them with PCF hydrogels to generate PCF‐FAMP. The constructed PCF‐FAMP as an injectable drug enabled fluorescence imaging monitoring of tumors. PCF‐FAMP was demonstrated to produce ·OH and ^1^O_2_ by in vitro experiments, and it induced strong ROS and heat generation upon near IR laser activation. The nanomedicine successfully induced ICD in vivo and successfully inhibited tumor growth in combination with ICB. Compared with conventional anti‐cancer drug delivery systems, the platform proposed in this study provided a new solution for tumor treatment taking the advantage of ICD and ICB multi‐pathway therapy, stable drug delivery, and real‐time monitoring. The application of fluorescence imaging and MRI‐assisted monitoring technology enabled the visualization of the treatment progress and response, providing valuable information for clinical decision‐making. These results indicated that the “multifunctional integration” strategy using hydrogels as carriers provided excellent performance. The better biocompatibility of hydrogels also endows them with great potential for real‐world applications in biological matrices.

## 4 Experimental Section

### Materials

Anhydrous ferric chloride (97%) was purchased from Shanghai Hu‐Test Laboratory Equipment Co. Manganese triacetate dihydrate (98%), N‐hydroxythiosuccinimide (sulfo‐NHS, 98%) was obtained from Shanghai Bidet Pharmaceutical Technology Co. (Shanghai, China). Poly(vinyl alcohol (solubility: 88–89 mol, viscosity: 3.2–3.8 mPa.s), MB (98%), poly(ethylene glycol) amino folic acid (FA‐PEG‐NH_2_, 97%), PEI (99%), chloroauric acid (48%−50% Au), TCPP (97%), zinc chloride (98%), PDA (98%), maleic anhydride (99%), polyphosphoric acid (phosphorus pentoxide, content % ≥ 85%), 1‐ethyl‐(3‐dimethylaminopropyl) carbodiimide hydrochloride (98.5%), and DPBF (97%) were purchased from Shanghai McLean Biochemical Science and Technology Co Ltd (Shanghai, China). Sodium citrate (98%) and sodium borohydride (98%) were supplied by Shanghai Aladdin Biochemistry Science and Technology Co Ltd (Shanghai, China). Ten percent fetal bovine serum, 1% penicillin‐streptomycin, Roswell Park Memorial Institute (RPMI)−1640 medium (with double antibiotic), and Dulbecco's Modified Eagle Medium (DMEM) (high glucose) medium were purchased from Jiangsu KGI Technology Co. Ltd (Nanjing, China). Annexin V‐FITC/PI Double Staining Apoptosis Detection Kit was obtained Shanghai Pebble Bio‐technology Company (Shanghai, China). Methyl thiazolyl tetrazolium (MTT) was purchased from Sangyo Bioengineering (Shanghai) Co. Ltd (Shanghai, China). HSP90, HSP90, CRT, and HMGB‐1 ELISA kits were bought from Beijing Bomax Technology Development Co. ATP assay kit was purchased from Biyuntian Biotechnology (Shanghai, China). Anti‐CD4‐FITC (11‐0041‐82), Anti‐CD8‐PE (12‐0081‐82), Anti‐CD3‐APC (17‐0032‐82) were purchased from Thermo Fisher Scientific (Massachusetts, USA). Ultrapure water was used throughout the work (resistance of 18.2 MΩ · cm).

### Synthesis of Fe_3_O_4_

Initially, 5.28 g of ferrous sulfate hexahydrate and 4.6 g of ferric chloride were dissolved in a 20 mL hydrochloric acid solution with a pH of 2. The solution was heated to 40 °C while undergoing mechanical stirring. Subsequently, the mixture was purged with N_2_ gas for 30 min. Following this, 10 mL of ammonium hydroxide was added dropwise, and the sample was stirred for 2 h at 85 °C. The last step involved magnetic separation, followed by washing with water and drying in an oven at 60 °C, to obtain Fe_3_O_4_.

### Synthesis of Fe_3_O_4_@Au

Five hundred milligrams of Fe_3_O_4_ were weighed and dissolved in a mixture comprising 10 mL of water, 10 mL of ethanol, and 10 mL of hydrochloric acid (0.2 m). The solution underwent ultrasonic mixing for 30 min, followed by magnetic separation. Subsequently, 10 mL of ethanol and 10 mL of hydrochloric acid (0.2 m) were added, and ultrasonic mixing was performed for an additional 30 min. After another round of magnetic separation, 1 mL of PEI and 9 mL of water were added, and the mixture was ultrasonically mixed for 30 min, followed by magnetic separation to give the final product, modified Fe_3_O_4_.

After that, 68 mg of chloroauric acid trihydrate were dissolved in 10 mL of water. Sodium hydroxide (2 m) was then added dropwise until the solution reached a pH of 8.5, resulting in the formation of the chloroauric acid solution. The magnetically recovered Fe_3_O_4_ was added to the solution, heated to 75 °C, and supplemented with 6 mL of sodium citrate solution (38.8 mm) and 4 mL of sodium borohydride solution (38.8 mm). The reaction proceeded for 5 min, followed by washing through magnetic recovery, and the final product, Fe_3_O_4_@Au, was dried at 60 °C in an oven.

### Preparation of FAM^x^P

First, 40 mg of TCPP was dissolved in a 10 mL of Tris buffer solution (pH = 8.5) to obtain a solution. Then, 500 mg of Fe_3_O_4_@Au and 8 mL of the TCPP solution were mixed together. Subsequently, 2 mL of 75 mm zinc chloride or manganese triacetate dihydrate solution was introduced, and the mixture underwent a 1  h reaction at 4 °C under dark conditions. The mixture solution was made to undergo magnetic recovery and washing, followed by freeze–drying, yielding FAZ and FAM.

Subsequently, 100 mg each of FAZ and FAM were weighed, and ultrasonication was conducted for 1 h in a PDA solution with a concentration of 2 mg mL^−1^. Magnetic recovery and washing ensued, followed by freeze–drying, yielding FAZP and FAMP.

### Preparation of PCF hydrogels

First, 5 g of PVA was added to 85 mL of water. Then, the mixture was heated and stirred at 95 °C for 2 h. Subsequently, the solution was cooled to ambient temperature, and 5 g of maleic anhydride and 500 mg of polyphosphoric acid were added. After stirring for 1 h, 50 mg of FA‐PEG‐NH_2_ solution was introduced and the mixture stirred for an additional 30 min. Following this, 5 mL of 0.8 m sulfo‐NHS and 5 mL of 0.4 M 1‐(3‐dimethylaminopropyl)‐3‐ethylcarbodiimide were added, and the mixture was stirred at 25 °C for 4 h to obtain 5% PCF hydrogel.

### Preparation of PCF‐FAM^x^P

Initially, 6 mg of FAM^x^P was dissolved in 600 µL of PBS. Subsequently, 400 µL of PCF solution was added, and after ultrasonic mixing, the mixture was placed in a −25 °C refrigerator. After couples of freezing (8 h) and thawing (4 h), the injectable PCF‐FAMP was obtained after 3 cycles.

### Characterization Techniques

FT‐IR spectroscopy was conducted using an Alpha IR system (Brüker, Guangzhou, China). XRD patterns were obtained using Cu Kα (*λ* = 1.5418 Å) radiation on a Brüker D8 Advance instrument (Billerica, MA, USA). XPS measurements were carried out on an ESCALAB 250 Xi X‐ray photoelectron spectrometer (Thermo Fisher Scientific, Waltham, MA, USA). The morphology and structure of the materials were investigated using field emission SEM (SU 8020 ESEM, Hitachi, Tokyo, Japan) and TEM (Talos F 200 X, Thermo Fisher Scientific). High‐resolution TEM and elemental mapping were performed using a Tecnai G2 F30 S‐Twin TEM system (FEI, Hillsboro, OR, USA). Magnetic properties were tested using a vibrating sample magnetometer (VSM, Lakeshore, Woburn, MA, USA)

### Detection of ·OH and^1^O_2_

The generation of ·OH was quantified based on the efficiency of MB degradation, as determined through a Shimadzu UV‐2550 UV–visible spectrometer (Kyoto, Japan). For the experiments, 15 mL of a 2 mg L^−1^ MB solution was employed. Then, 100 µL of hydrogen peroxide was introduced, along with 25 mg of the specified nanomaterial (Fe_3_O_4_, Fe_3_O_4_@Au, FAZ, FAM, FAZP, and FAMP), and the mixture was subjected to a 2 h reaction for subsequent analysis.

The quantification of ^1^O_2_ production was conducted using DPBF. For analysis, 20 µL of DPBF solution (1 mg mL^−1^) was combined with 100 µL of hydrogen peroxide, and the resulting mixture was reacted with 25 mg of the specified nanomaterial for a duration of 2 h. The nanomaterials under investigation included Fe_3_O_4_, Fe_3_O_4_@Au, FAZ, FAM, FAZP and FAMP.

### Cell Culture

HeLa cell line was obtained from Servicebio (STCC10603P‐1, Wuhan, China). H8 cells were purchased from the Cell Resource Database of the Chinese Academy of Sciences (Shanghai, China). HeLa cells and H8 cells were cultured at 37 °C and 5% carbon dioxide in a humidified atmosphere and grown in a medium containing 10% fetal bovine serum and 1% penicillin–streptomycin in DMEM and RPMI‐1640.

### In Vitro Fluorescence Monitoring

FAM, FAZ, FAMP, and FAZP were each prepared as 6 mg mL^−1^ solutions, and 200 µL of each of the solution was taken into H8 cells containing 2 mL of medium. The cells were washed with PBS after 48 h of incubation, and bright field and fluorescence images were taken using a research‐grade Olympus IX73 inverted microscope (Yijingtong Optical Science and Technology (Shanghai) Co., Ltd., Shanghai, China).

In order to study the effect of PDA concentration on drug release, PDA solutions with concentrations of 1, 2 and 3 mg mL^−1^ were added to FAM. FAMP‐1, FAMP‐2, and FAMP‐3 were obtained according to the above steps, and the obtained materials were formulated into a 6 mg mL^−1^ solution, respectively. Two hundred microliters of each of the solutions were placed into HeLa cells containing 2 mL of medium, and the cells were washed with PBS after 24 h of incubation, and the bright‐field and fluorescence images were captured using the inverted microscope.

Fe_3_O_4_, Fe_3_O_4_@Au, FAM, FAZ, FAMP, and FAZP were each prepared as solutions of 6 mg mL^−1^ in water, and 200 µL were placed into HeLa cells containing 2 mL of medium. The cells were incubated for 0, 24, and 48 h. Cells were washed with PBS, and bright‐field and fluorescence images were captured using the inverted microscope.

### Apoptosis Assay

HeLa cell apoptosis induced by different materials was detected using the Annexin V‐FITC/PI Double Stain Apoptosis Detection Kits. HeLa cells were cultured in Petri dishes and divided into six groups: (I) Control, no pre‐treatment was done in this group; (II) laser irradiation for 10 min; (III) FAZP was prepared as a 6 mg mL^−1^ solution, and 200 µL of the solution were taken into HeLa cells containing 2 mL of medium and incubated for 12 h; (IV) FAMP was prepared as a 6 mg mL^−1^ solution, 200 µL were taken into HeLa cells containing 2 mL of medium into HeLa cells containing 2 mL of medium and incubated for 12 h; (V) 10 min of irradiation immediately after the addition of FAZP material, followed by 12 h of incubation; and (VI) 10 min of irradiation immediately after the addition of FAMP material, followed by 12 h of incubation. Subsequently, the cells were washed and collected, resuspended in PBS buffer, and followed up according to the kit instructions. The cells were analyzed and detected using a flow cytometer FACSVerse (Biddy Medical Devices (Shanghai) Co. (Shanghai, China).

### In Vitro Cytotoxicity Assay

Cytotoxicity was measured using an MTT assay. HeLa cells were cultured in Petri dishes and divided into six groups: I) Control, no pretreatment; II) laser irradiation for 10 min; III) FAZP was prepared as a 6 mg mL^−1^ solution, and 200 µL of the above solution were taken into HeLa cells containing 2 mL of culture medium, and incubated for 12 h; IV) FAMP was prepared as a 6 mg mL^−1^ solution, and 200 µL were taken into HeLa cells containing 2 mL of medium into HeLa cells and incubated for 12 h; V)FAZP was added, and immediately irradiated for 10 min, followed by incubation for 12 h; and VI) FAMP was added and immediately irradiated for 10 min, followed by incubation for 12 h. The HeLa cells and H8 cells at the end of the incubation were inoculated at a concentration of 10^4^ cells per well into 100 µL of fresh cell culture medium in 96‐well plates. Then, 10 µL of MTT (0.5 mg mL^−1^) were added to each well and the samples were incubated at 37 °C for 4 h. Subsequently, the medium in each well was replaced with 100 µL of methoxazole solubilization solution. After incubation for 10 min, the absorbance at 570 nm was measured using an enzyme marker (SpectraMax i3, Meigu Molecular Instruments (Shanghai) Co. (Shanghai, China).

### In Vitro Measurement of IICD

In order to verify the effect of nanomedicine‐induced ICD in HeLa cells, the pre‐ and post‐treatment changes of intracellular GSH, HSP90, CRT, HMGB1, and ATP content were examined. There were six groups: I) Control, no pretreatment; II) laser irradiation for 10 min; III) FAZP was formulated into a 6 mg mL^−1^ solution, and 200 µL were placed into HeLa cells containing 2 mL of culture medium with incubation for 12 h; IV) FAMP was formulated into a 6 mg mL^−1^ solution, and 200 µL were placed into HeLa cells containing 2 mL of medium into HeLa cells with incubation for 12 h; V) FAZP was added and immediately irradiated for 10 min, followed by incubation for 12 h; and VI) FAMP was added and immediately irradiated for 10 min, followed by incubation for 12 h. The HeLa cells and H8 cells at the end of the incubation were inoculated at a concentration of 10^4^ cells per well into 100 µL of fresh cell culture medium in 96‐well plates. At the end of incubation, GSH, HSP90, CRT, and HMGB‐1 were detected using ELISA kits (Jiangsu No Enzyme Industry Co., Ltd., Taixing, China) and ATP assay kits (Biyuntian Biotechnology Co., Ltd., Shanghai, China) according to the manufacturers’ instructions.

### Detection of Intracellular ROS/JC‐1

In order to verify the apoptotic effect of nanomedicine‐induced HeLa cells, the pre‐ and post‐treatment changes of intracellular ROS/JC‐1 content were evaluated. Samples were divided into six groups: I) Control, no pretreatment; II) laser irradiation for 10 min; III) FAZP was formulated into a 6 mg mL^−1^ solution, and 200 µL were placed into HeLa cells containing 2 mL of culture medium with incubation for 12 h; IV) FAMP was formulated into a 6 mg mL^−1^ solution, and 200 µL were put into HeLa cells containing 2 mL of medium into HeLa cells with incubation for 12 h; V) 10 min of irradiation immediately after the addition of FAZP material, followed by 12 h of incubation; and VI) 10 min of irradiation immediately after the addition of FAMP material, followed by 12 h of incubation. Next, using the reactive oxygen species detection kit (Beijing Priority Genetics Co., Ltd., Beijing, China), all the cells were stained with DCFH‐DA staining to detect ROS. Next, using the Mitochondrial Membrane Potential Assay Kit (JC‐1, Beijing Solepol Technology Co., Ltd., Beijing, China), and all cells were stained with JC‐1 dye to observe the changes in MMP. Cells were collected for FCM analysis.

### In Vivo Antitumor Therapy in Mice

Female BALB/c nude mice were purchased from Yangzhou University, China, and housed in Nanjing Pusheng Biomedical Technology Co. (Nanjing, China). The tumor system was divided into five groups: I) Control; II) PCF‐FAMP; III) PCF‐FAMP + laser (1.0 W cm^−2^); IV) A‐PD‐L1; and V) PCF‐FAMP + laser (1.0 W cm^−2^) + A‐PD‐L1. Group (II), group (III), and group (V) were injected in situ at the tumor site with 100 µL of PCF‐FAMP drug (2% PCF hydrogel with 6 mg mL^−1^ of FAMP). Groups (III) and (V) were administered with laser irradiation for 10 min on the first and second day. In addition, Group (IV) and group (V) were injected with A‐PD‐L1 (1 mg k^−1^g, in 200 µL of PBS for each mouse) on day 1. The treatment ended after 10 days. During the 10‐day observation period, weight and tumor diameter were recorded alternately. The temperature and infrared thermal images of each group set were collected using a thermal imager.

The tumor‐bearing mice were sacrificed 10 days after injection; tumors and major organs were collected from one mouse in each group, and histologic evaluation was performed with H&E staining. Tumor proliferation was assessed using PCNA and TUNEL staining.

Mice were sacrificed on day 10 to obtain blood samples. Serum was collected from each mouse, and levels of interleukin (IL)−6, IL‐12, TNF‐α, and IFN‐γ were measured by ELISA. Proximal tumors were collected from all groups on day 10. Finally, single‐cell suspensions were stained with anti‐CD3‐APC, anti‐CD8‐PE, and anti‐CD4‐FITC, for FCM analysis. The right (distant) tumor was also collected, and immunofluorescence staining of CD4^+^ and CD8^+^ *T* cells was performed on the tissue sections.

To evaluate the in vivo toxicity of the PCF‐FAMP group as in the previous step, all major organs and blood samples were collected for testing on the tenth day.

Relevant manipulations concerning animal studies complied with the guidelines of the Institutional Animal Care Use Committee and were approved by the Ethics Committee of the University (202404A034). All mice were housed under specific pathogen‐free conditions with a 12 h light–dark cycle and had access to food and water ad libitum.

### Bilateral Intra‐Tumoral Fluorescence Profiles and Nuclear Magnetic Imaging Measurements in Mouse Tumors

One hundred milliliters of PCF‐FAMP+A‐PD‐L1 were injected into mice at the tumor tissue. Fluorescence photographs were taken before drug injection, after drug injection, 10 min of laser irradiation after drug injection, and 2, 12, and 48 h after laser irradiation. MRI measurements were performed of the mice into which the drugs were introduced. The corresponding T 1 relaxation times were obtained to assess the ability of MRI to capture accurate images of FAMP in vitro and in vivo. MRI was acquired from two time points, pre‐dose and 48 h post‐dose.

### Statistical Analysis

All the data are shown as mean ± SD from at least three experiments (*n* = 3). Statistical data was analyzed through one‐way variance for comparison among multiple groups or two‐tail paired Student's *t*‐test for comparison between two groups. ^*^ *p* <  0.05 was set as statistically significant (^**^ *p* < 0.01, ^***^ *p* < 0.001). All data were analyzed using GraphPad Prism 8.0.

## Conflict of Interest

The authors declare no conflict of interest.

## Supporting information

Supporting Information

## Data Availability

The data that support the findings of this study are available from the corresponding author upon reasonable request.

## References

[1] D. Zhang , Z. Lin , M. Wu , Z. Cai , Y. Zheng , L. He , Z. Li , J. Zhou , L. Sun , G. Chen , Y. Zeng , J. Li , J. Liu , H. Yang , X. Liu , Adv. Sci. 2021, 8, 2003504. 10.1002/advs.202003504 PMC7967047 33747739

[2] Q. Xiang , C. Yang , Y. Luo , F. Liu , J. Zheng , W. Liu , H. Ran , Y. Sun , J. Ren , Z. Wang , Small 2022, 18, 2107809. 10.1002/smll.202107809 35143709

[3] H. Tang , X. Xu , Y. Chen , H. Xin , T. Wan , B. Li , H. Pan , D. Li , Y. Ping , Adv. Mater. 2021, 33, 2006003. 10.1002/adma.202006003 33538047

[4] Z. Asadzadeh , E. Safarzadeh , S. Safaei , A. Baradaran , A. Mohammadi , K. Hajiasgharzadeh , A. Derakhshani , A. Argentiero , N. Silvestris , B. Baradaran , Cancers 2020, 12, 1047. 32340275 10.3390/cancers12041047 PMC7226590

[5] F. Zhou , B. Feng , H. Yu , D. Wang , T. Wang , Y. Ma , S. Wang , Y. Li , Adv. Mater. 2019, 31, 1805888. 10.1002/adma.201805888 30762908

[6] G. Hou , J. Qian , M. Guo , W. Xu , J. Wang , Y. Wang , A. Suo , Chem. Eng. J. 2022, 435, 134778.

[7] Y. Cheng , Y. D. Xia , Y. Q. Sun , Y. Wang , X. B. Yin , Adv. Mater. 2023, 36, 2308033. 10.1002/adma.202308033 37851918

[8] C. Liu , J. Xing , O. U. Akakuru , L. Luo , S. Sun , R. Zou , Z. Yu , Q. Fang , A. Wu , Nano Lett. 2019, 19, 5674. 31361142 10.1021/acs.nanolett.9b02253

[9] J. Mu , L. He , W. Fan , W. Tang , Z. Wang , C. Jiang , D. Zhang , Y. Liu , H. Deng , J. Zou , O. Jacobson , J. Qu , P. Huang , X. Chen , Small 2020, 16, 2004016. 10.1002/smll.202004016 32985099

[10] F. Zhang , K. Cheng , Z. Y. Huang , X. L. Hou , X. S. Zhang , Z. T. Zhong , Y. G. Hu , X. L. Lei , Y. Li , P. J. Zhang , Y. Zhao , Q. Xu , Adv. Funct. Mater. 2023, 33, 2212740.

[11] A. Z. Juthi , M. Aquib , M. A. Farooq , S. Ghayas , F. Khalid , G. F. Boafo , D. P. Wande , D. H. Khan , T. Z. Bithi , R. Bavi , B. Wang , Environ. Chem. Lett. 2020, 18, 1509.

[12] L. Zhai , Y. Shi , Y. Yan , A. Lu , X. Liu , L. Lei , Y. Sun , L. Jiang , X. Wang , H. Qian , J. Wang , Chin. Chem. Lett. 2023, 34, 108104.

[13] R. Tian , X. Qiu , W. Mu , B. Cai , Z. Liu , S. Liu , X. Chen , Chin. Chem. Lett. 2024, 35, 108343.

[14] A. S. Mikhail , R. Morhard , M. Mauda‐Havakuk , M. Kassin , A. Arrichiello , B. J. Wood , Adv. Drug. Deliver. Rev. 2023, 202, 115083. 10.1016/j.addr.2023.115083 PMC11616795 37673217

[15] Y. Sun , Q. Lu , D. Dong , R. Chen , Z. Chen , Z. Xie , H. Zhu , Q. Bu , H. He , S. Wang , Chem. Eng. J. 2024, 482, 149015.

[16] J. Yang , J. Qu , X. Teng , W. Zhu , Y. Xu , Y. Yang , X. Qian , Adv. Healthcare Mater. 2023, 12, 2301084. 10.1002/adhm.202301084 37219912

[17] R. Wang , L. Xing , Y. Ha , P. Zhong , Z. Wang , Y. Cao , Z. Li , Solar. Rrl. 2023, 7, 2300269.

[18] B. Liu , X. Gu , Q. Sun , S. Jiang , J. Sun , K. Liu , F. Wang , Y. Wei , Adv. Funct. Mater. 2021, 31, 2010779.

[19] W. Cheng , J. Nie , L. Xu , C. Liang , Y. Peng , G. Liu , T. Wang , L. Mei , L. Huang , X. Zeng , ACS Appl. Mater. Interfaces 2017, 9, 18462. 28497681 10.1021/acsami.7b02457

[20] J. Zhang , Y. Ji , P. Wang , Q. Shao , Y. Li , X. Huang , Adv. Funct. Mater. 2019, 30, 1906579.

[21] S. Sau , S. Pandit , S. Kundu , Surf. Interfaces 2021, 25, 101198.

[22] X. Miao , Z. Li , K. Hou , Q. Gao , Y. Huang , J. Wang , S. Yang , Chem. Eng. J. 2023, 476, 146848.

[23] A. J. Siddiqa , K. Chaudhury , B. Adhikari , Colloid. Surface. B. 2014, 116, 169. 10.1016/j.colsurfb.2013.12.040 24463149

[24] T. K. Ryu , S. W. Baek , R. H. Kang , S. W. Choi , Adv. Funct. Mater. 2016, 26, 6428.

[25] H. Hou , X. Huang , G. Wei , F. Xu , Y. Wang , S. Zhou , ACS Appl. Mater. Interfaces 2019, 11, 29579. 31359756 10.1021/acsami.9b09671

[26] Y. Liu , W. Jin , Y. Zhao , G. Zhang , W. Zhang , Appl Catal B 2017, 206, 642.

[27] S. Yu , H. Yu , P. Si , Z. Wang , B. Wang , W. Lin , J. Mater. Chem. B 2022, 10, 8988. 36314257 10.1039/d2tb01619b

[28] X. Xu , J. Ma , A. Wang , N. N.‐S Zheng , Chem. Sci. 2024, 15, 1769. 38303932 10.1039/d3sc05504c PMC10829015

[29] T. Wang , H. Liu , X. Wang , L. Tang , J. Zhou , X. Song , L. Lv , W. Chen , Y. Chen , X. Li , ACS Catal. 2023, 13, 13902.

[30] W. Wang , C. Hao , M. Sun , L. Xu , C. Xu , H. Kuang , Adv. Funct. Mater. 2018, 28, 1800310.

[31] X. Zhang , C. Liu , Y. Lyu , N. Xing , J. Li , K. Song , X. Yan , J. Colloid. Interf. Sci. 2023, 648, 457. 10.1016/j.jcis.2023.05.172 37302229

[32] H. Zhang , X. T. Tian , Y. Shang , Y. H. Li , X. B. Yin , ACS Appl. Mater. Interfaces 2018, 10, 28390. 30066560 10.1021/acsami.8b09680

[33] T. J. Zhou , X. Wan , M. M. Zhang , D. M. Liu , L. L. Huang , L. Xing , Y. Wang , H. L. Jiang , Biomaterials 2023, 300, 122205. 37348324 10.1016/j.biomaterials.2023.122205

[34] S. S. Wan , Q. Cheng , X. Zeng , X. Z. Zhang , ACS Nano 2019, 13, 6561. 31136707 10.1021/acsnano.9b00300

[35] Z. Chu , J. Yang , W. Zheng , J. Sun , W. Wang , H. Qian , Coordin. Chem. Rev. 2023, 481, 215049.

[36] J. P. Luzio , P. R. Pryor , N. A. Bright , Lysosomes: Fusion and Function 2007, 8, 622. 10.1038/nrm2217 17637737

[37] X. Gao , K. Bao , Y. Zhang , L. Liu , Y. Li , C. Hu , Y. Zhao , W. Lu , X. Wei , Adv. Funct. Mater. 2023, 33, 2215014.

[38] S. D. Jeong , B. K. Jung , H. M. Ahn , D. Lee , J. Ha , I. Noh , C. O. Yun , Y. C. Kim , Adv. Sci. 2021, 8, 2001308. 10.1002/advs.202001308 PMC8025002 33854870

[39] A. Ahmed , S. W. G. Tait , Mol. Oncol. 2020, 14, 2994. 33179413 10.1002/1878-0261.12851 PMC7718954

[40] P. Song , B. Wang , Q. Pan , T. Jiang , X. Chen , M. Zhang , J. Tao , X. Zhao , Carbohydr Polym 2023, 312, 120837. 37059562 10.1016/j.carbpol.2023.120837

[41] X. Liu , J. Jiang , Y. P. Liao , I. Tang , E. Zheng , W. Qiu , M. Lin , X. Wang , Y. Ji , K. C. Mei , Q. Liu , C. Chang , Z. Wainberg , A. Nel , H. Meng , Adv. Sci. 2021, 8, 2002147. 10.1002/advs.202002147 PMC7967046 33747719

[42] F. H. Schopf , M. M. Biebl , J. Buchner , Nat. Rev. Mol. Cell Biol. 2017, 18, 345. 28429788 10.1038/nrm.2017.20

[43] Z. Dai , J. Tang , Z. Gu , Y. Wang , Y. Yang , Y. Yang , C. Yu , Nano Lett. 2020, 20, 6246. 32786942 10.1021/acs.nanolett.0c00713

[44] Y. Chen , J. Wang , Q. Wang , X. Lei , J. Zhao , Z. Xu , J. Ming , ACS Sustainable Chem. Eng. 2023, 11, 11745.

[45] N. Yao , Y. J. Li , Y. H. Lei , N. Hu , W. M. Chen , Z. Yao , M. Yu , J. S. Liu , W. C. Ye , D. M. Zhang , J. Exp. Clin. Canc. Res. 2016, 35, 192. 10.1186/s13046-016-0457-1 PMC5146873 27931237

[46] J. Wan , X. Zhang , Z. Li , F. Mo , D. Tang , H. Xiao , J. Wang , G. Rong , T. Liu , Adv. Healthcare Mater. 2023, 12, 2202710. 10.1002/adhm.202202710 36527737

[47] Y. Xu , Y. Guo , C. Zhang , M. Zhan , L. Jia , S. Song , C. Jiang , M. Shen , X. Shi , ACS Nano 2022, 16, 984. 35023715 10.1021/acsnano.1c08585

[48] L. Zhang , S. Tong , Q. Zhang , G. Bao , ACS Appl. Nano Mater. 2020, 3, 6785.

[49] H. Chen , F. Wu , X. Xie , W. Wang , Q. Li , L. Tu , B. Li , X. Kong , Y. Chang , ACS Nano 2021, 15, 20643. 34878760 10.1021/acsnano.1c09635

[50] Z. Li , Z. Chu , J. Yang , H. Qian , J. Xu , B. Chen , T. Tian , H. Chen , Y. Xu , F. Wang , ACS Nano 2022, 16, 15471. 35981098 10.1021/acsnano.2c08013

[51] R. Wang , M. Qiu , L. Zhang , M. Sui , L. Xiao , Q. Yu , C. Ye , S. Chen , X. Zhou , Adv Mater 2023, 35, 2306748. 10.1002/adma.202306748 37689996

[52] D. J. Propper , F. R. Balkwill , Nat. Rev. Clin. Oncol. 2022, 19, 237. 34997230 10.1038/s41571-021-00588-9

[53] H. Wang , Z. Gao , D. Jiao , Y. Zhang , J. Zhang , T. Wang , Y. Huang , D. Zheng , J. Hou , D. Ding , W. Zhang , Adv. Funct. Mater. 2023, 33, 2214499.

[54] W. Ni , J. Wu , H. Fang , Y. Feng , Y. Hu , L. Lin , J. Chen , F. Chen , H. Tian , Nano Lett. 2021, 21, 7796. 34516141 10.1021/acs.nanolett.1c02782

[55] M. Negi , N. Kaushik , L. N. Nguyen , E. H. Choi , N. K. Kaushik , Free Radical Bio. Med. 2023, 201, 26. 36907254 10.1016/j.freeradbiomed.2023.03.009

