# Supplementary material for: Targeting Hydrogel for Intelligent Recognition and Spatiotemporal Control in Cell‐Based Therapeutics
Source: Adv Sci (Weinh). 2024 Jun 14;11(30):2404172. doi: 10.1002/advs.202404172 (PMC11321622; doi:10.1002/advs.202404172)
Supplement: Supplementary file 1 — Supporting Information [file ADVS-11-2404172-s001.docx]

**Supporting Information**

**Targeting hydrogel for intelligent recognition and spatiotemporal control in cell-based therapeutics**

Weilin Hou,^1,a^ Wei Mao,^1,2,a^ Jun Sun,^1,3,a^ Zhiqiang Liu,^1^ Wei Shen,^1,^* Hian Kee Lee,^1,4,^* Sheng Tang^1,^*

^1^School of Environmental and Chemical Engineering, Jiangsu University of Science and Technology, Zhenjiang, 212003, Jiangsu Province, PR China

^2^Central-Southern Safety and Environmental Technology Institute Co. Ltd., Wuhan 430071, PR China

^3^School of Chemistry, The University of New South Wales, Sydney, NSW 2052, Australia

^4^Department of Chemistry, National University of Singapore, 3 Science Drive 3, Singapore, 117543, Singapore

^a^W. Hou, W. Mao and J. Sun contribute equally as co-first authors.

*Corresponding authors’ email addresses: shenweivv@126.com (W. Shen); chmleehk@nus.edu.sg (H. K. Lee); tangsheng.nju@gmail.com (S. Tang)

1. **In vitro photothermal properties**

The optical properties of FAMP (6 mg, with added 1 mL of H_2_O) were investigated using UV-visible spectroscopy. Photothermal properties (808 nm (1.0 W/cm^2^) laser irradiation) were also evaluated at concentrations of 1, 2, 4 and 6 mg/mL. PBS was used as a control solution. Thermal images were taken with a thermal imaging camera FOTRIC 285 (Shanghai Thermal Imaging Technology Co., Ltd., Shanghai, China).

1. **Fluorescence change of M^x^-TCPP**

TCPP, Zn-TCPP and Mn-TCPP were each prepared as 10 mg/mL aqueous solutions, and the fluorescence of the solutions (λ_ex_ = 415 nm) was measured using FS5 fluorescence spectroscopy (Edinburgh Instruments, Kirkton Campus, U.K.). GSH (3mM) was added to the Zn-TCPP and Mn-TCPP solutions separately and the fluorescence changes were measured in the same way.

1. **Physical drawing of PCF-FAMP and its thermal imaging and extrusion experiments**

The 5 % PCF-FAMP hydrogels were diluted to 1 %, 2 % and 3 % by adding ultrapure water, and 6 mg of FAMP were added to each of them. The gels were subjected to freezing in a refrigerator at -20 ℃ and thawed at ambient temperature for cycling, several times. Subsequently, the 1 %, 2 %, and 3 % PCF-FAMP hydrogels were heated in a water bath to 25 ℃ and 45 ℃, and were photographed normally and with a thermal imager. A 2 % hydrogel was aspirated with a 2 mL syringe and photographed in the squeezed state.

1. **Rheological testing of PCF-FAMP**

The rheological behavior of PCF-FAMP was recorded on a rheometer (Discovery HR-10, TA Instruments Waters, New Castle, DE, USA). The temperatures were at 25 °C and 40 °C, respectively, with frequency sweeps from 0.1 to 50 Hz at a constant strain of 1 %. Simultaneous temperature scans were performed from 25 °C~ 80 °C at a constant strain of 1 %, to test the stability of the hydrogel.


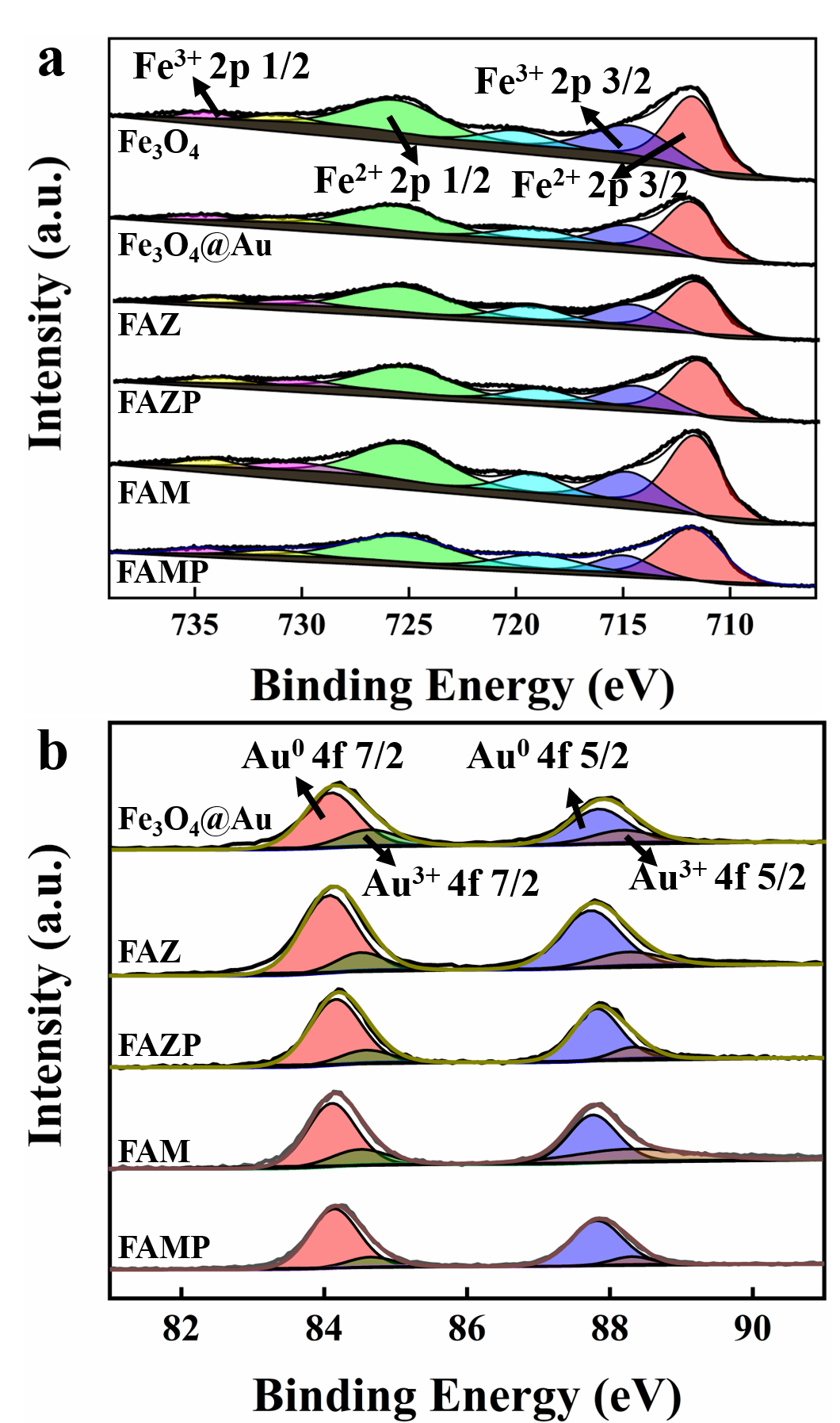


**Figure S1.** XPS spectra of (a) Fe 2p; (b) Au 4f.


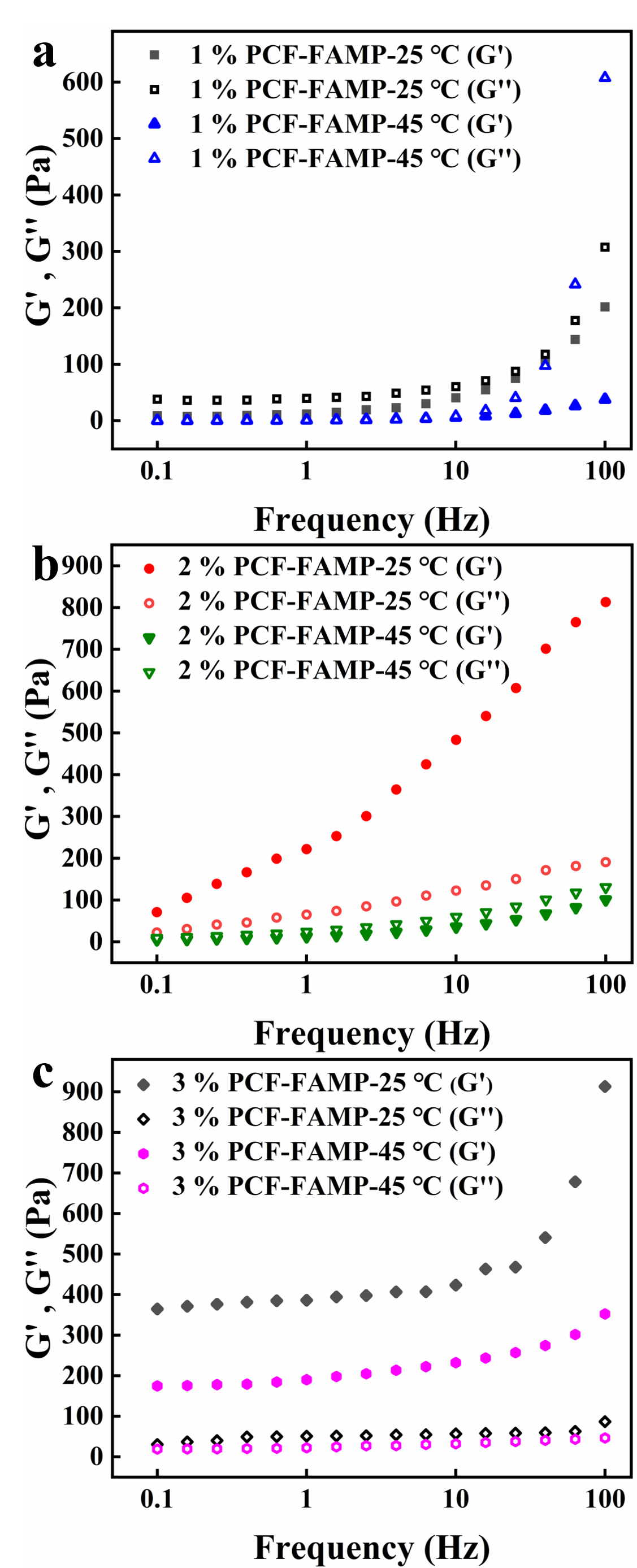


**Figure S2.** (a)-(b) Frequency sweep analysis of hydrogel with PCF-FAMP concentrations of 1 %, 2 % and 3 % at 25 °C and 45 °C.


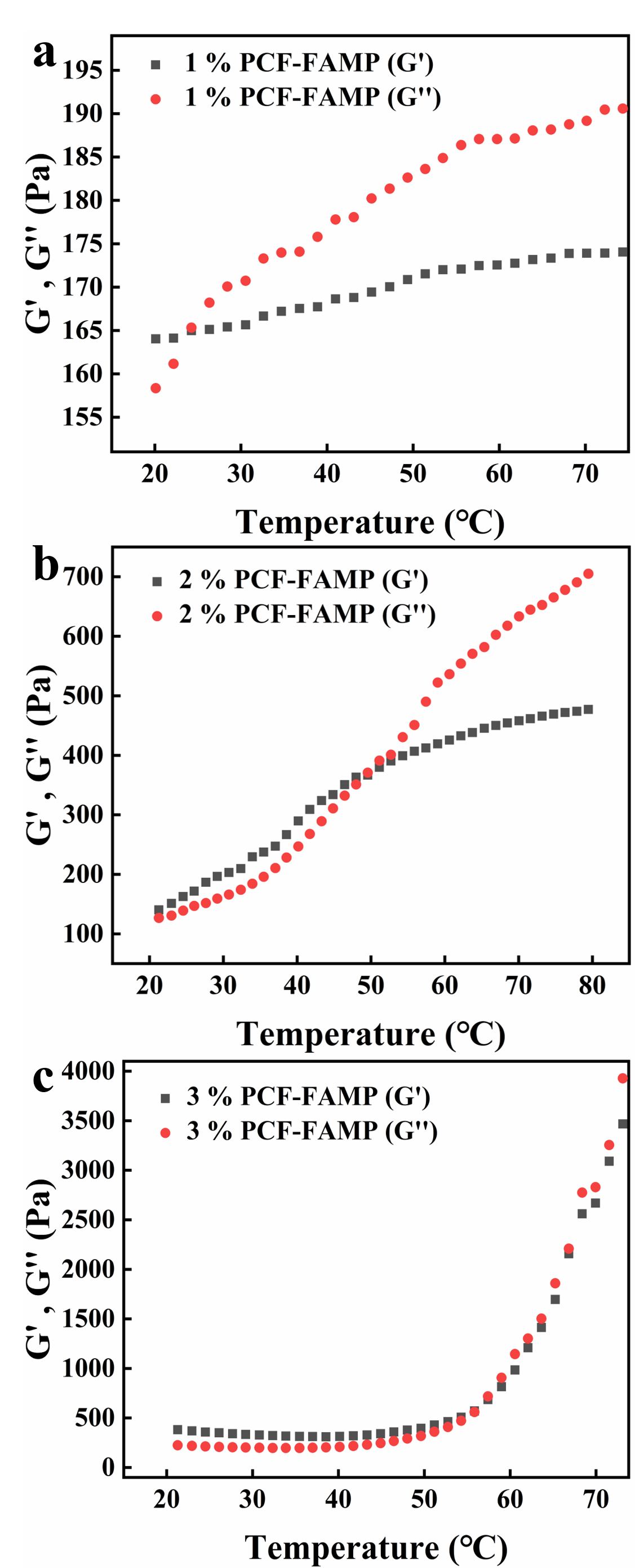


**Figure S3.** (a)-(b) Temperature sweep test of hydrogel with PCF-FAMP concentrations of 1 %, 2 % and 3 %.


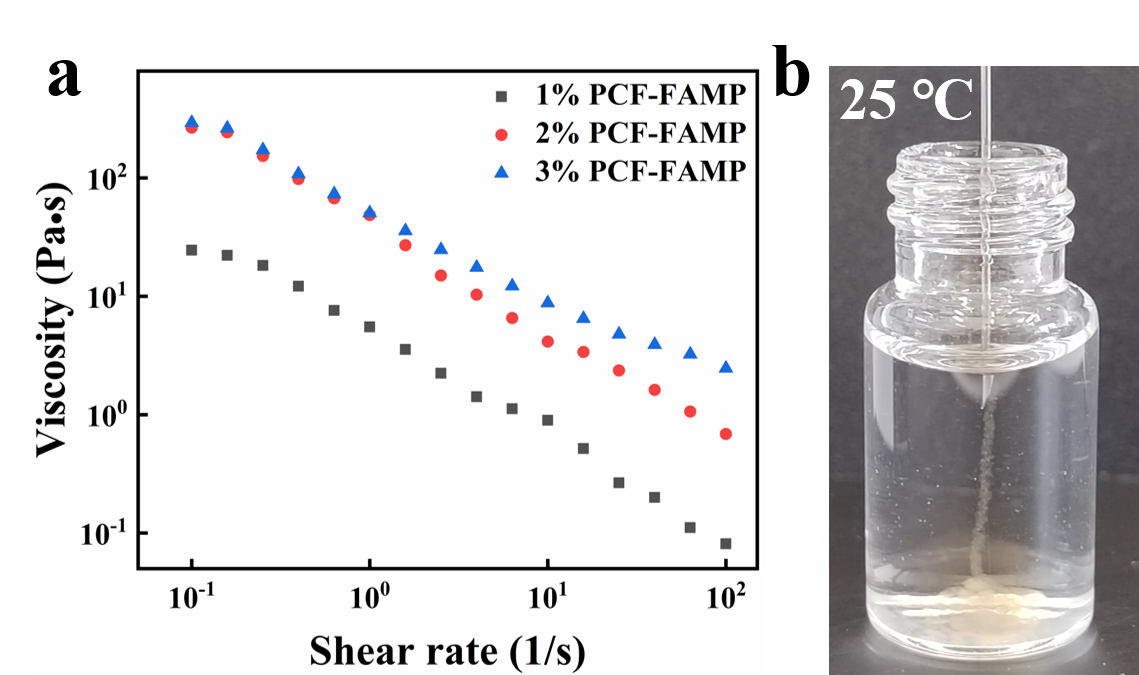


**Figure S4.** (a) Rheological analysis of hydrogel with PCF-FAMP concentrations of 1 %, 2 % and 3 % in shear-thinning behaviors; (b) Photograph depicting injection of 2% PCF-FAMP hydrogel into an aqueous solution.


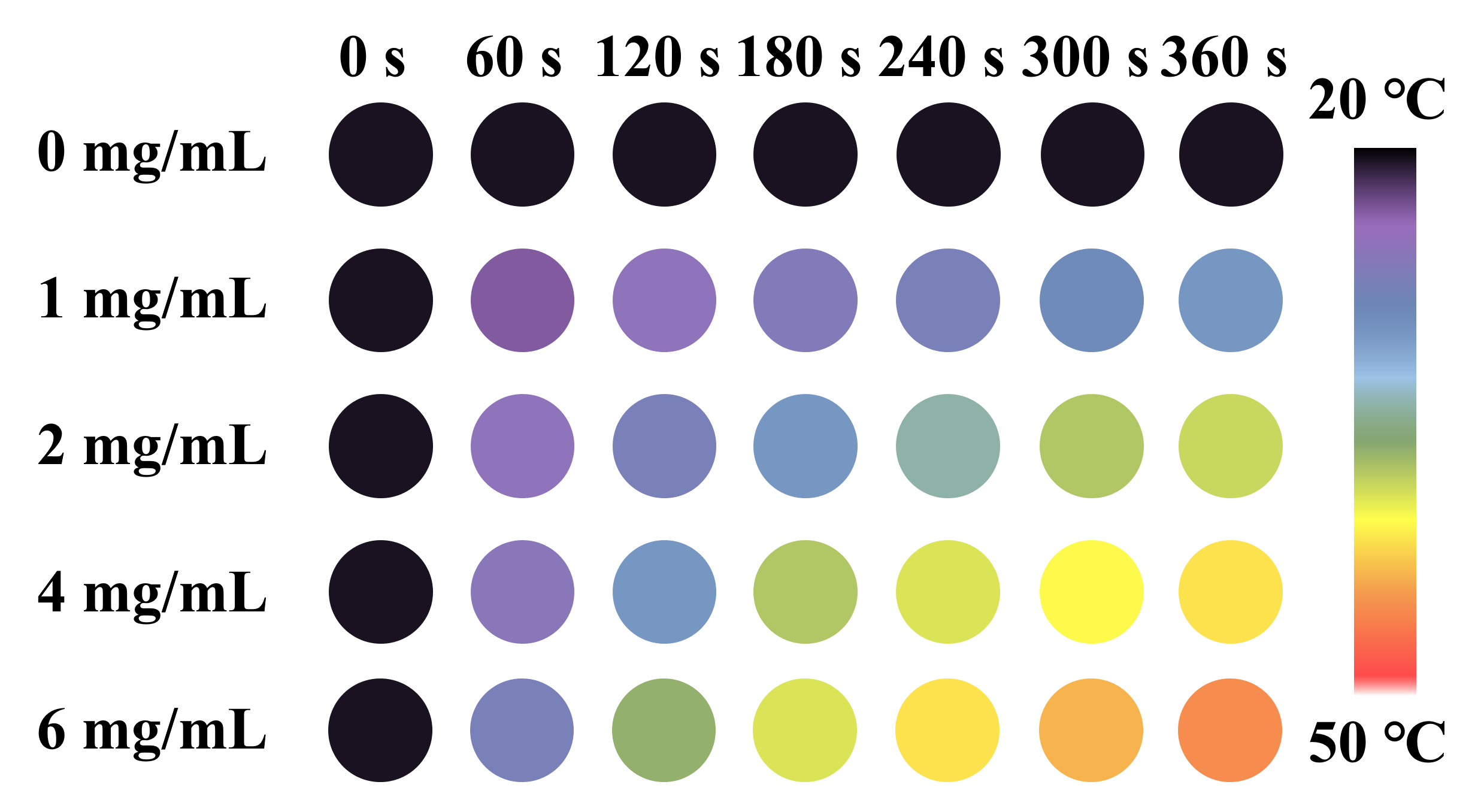


**Figure S5.** Infrared thermal images of different concentrations of PBS and FAMP aqueous suspensions under NIR irradiation (808 nm, 1.0 W/cm^2^).


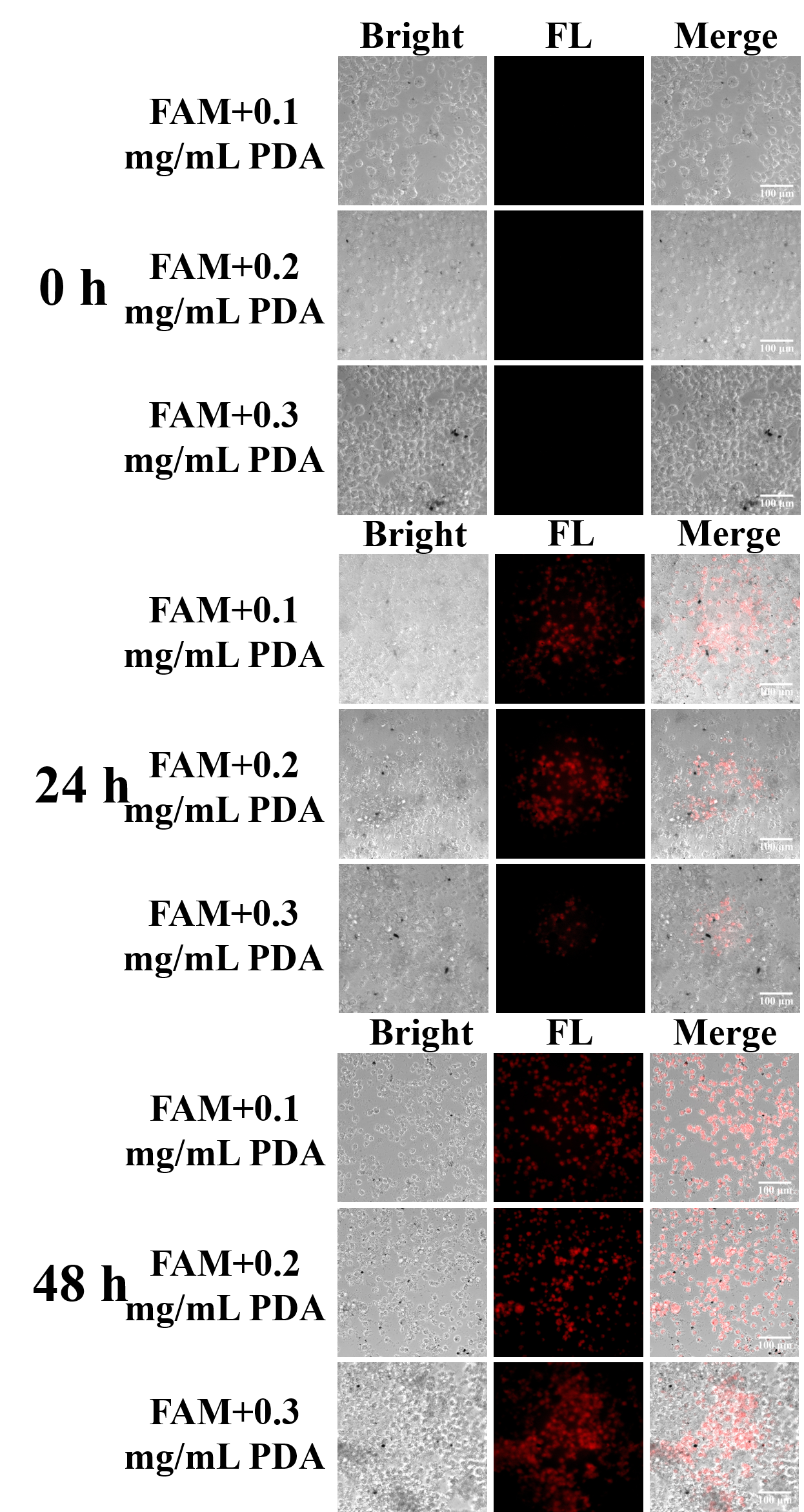


**Figure S6.** Bright-field images, FL (fluorescence) images and merge images of FAM modified with different concentrations of PDA incubated with HeLa cells for 0 h, 24 h and 48 h.


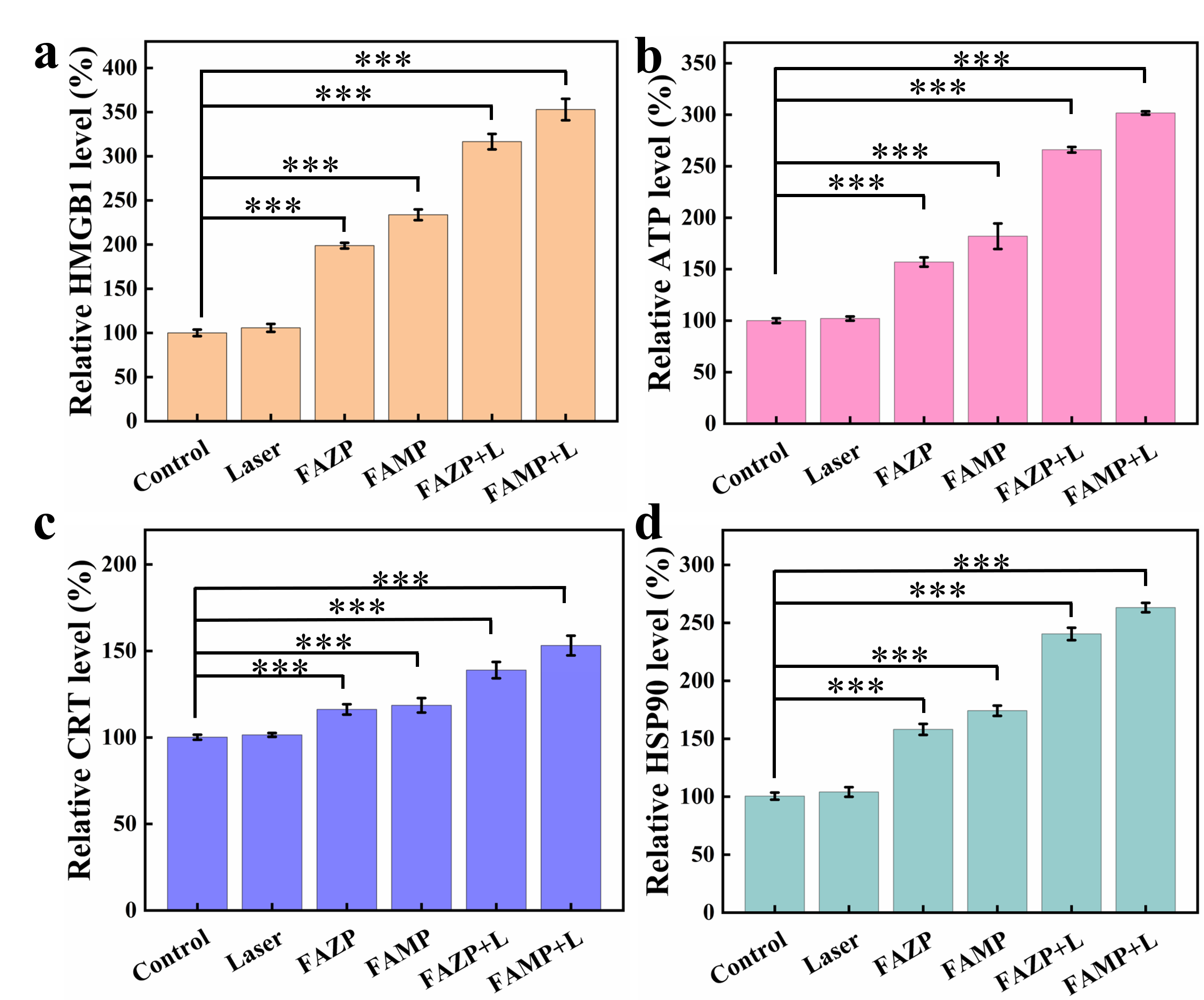


**Figure S7.** (a)-(d) The release levels of HMGB1, ATP, CRT and HSP90 after different treatments. Data are represented as mean ± SD (n = 5). Student's t-test, * P < 0.05, ** P < 0.01, *** P < 0.001.


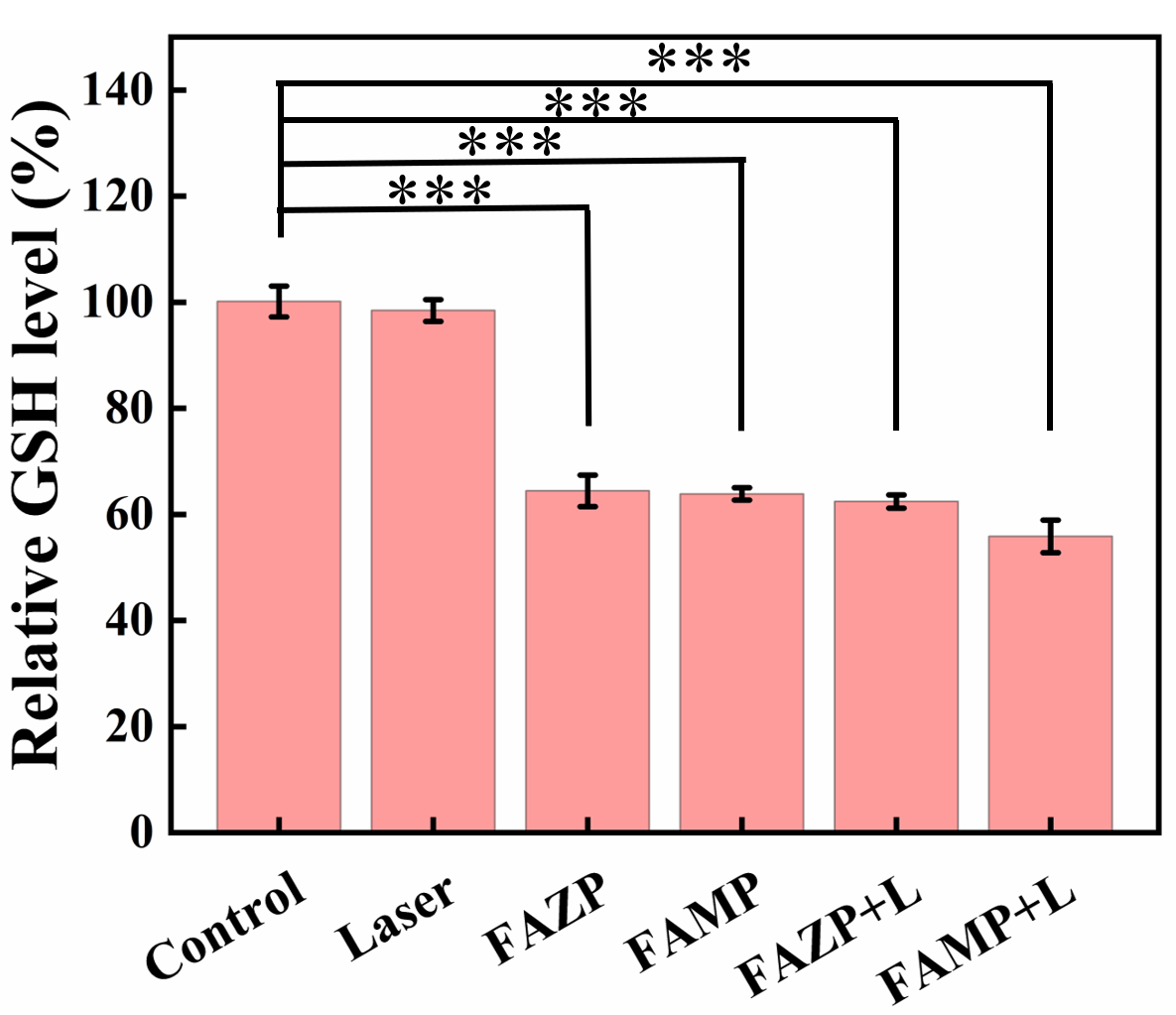


**Figure S8.** Intracellular GSH levels after different treatments. Data are represented as mean ± SD (n = 5). Student's t-test, * P < 0.05, ** P < 0.01, *** P < 0.001.


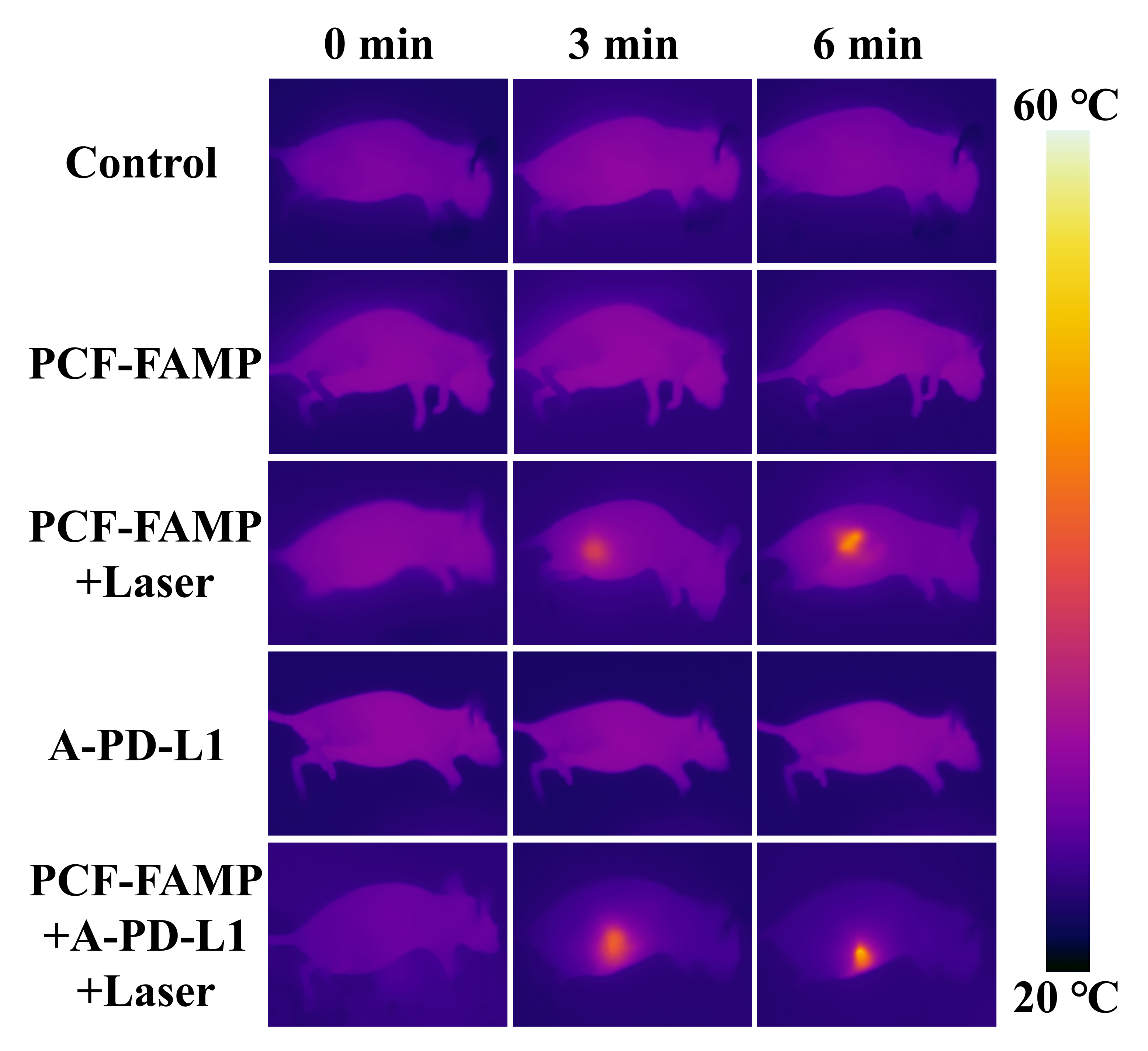


**Figure S9.** Thermal images of mice.


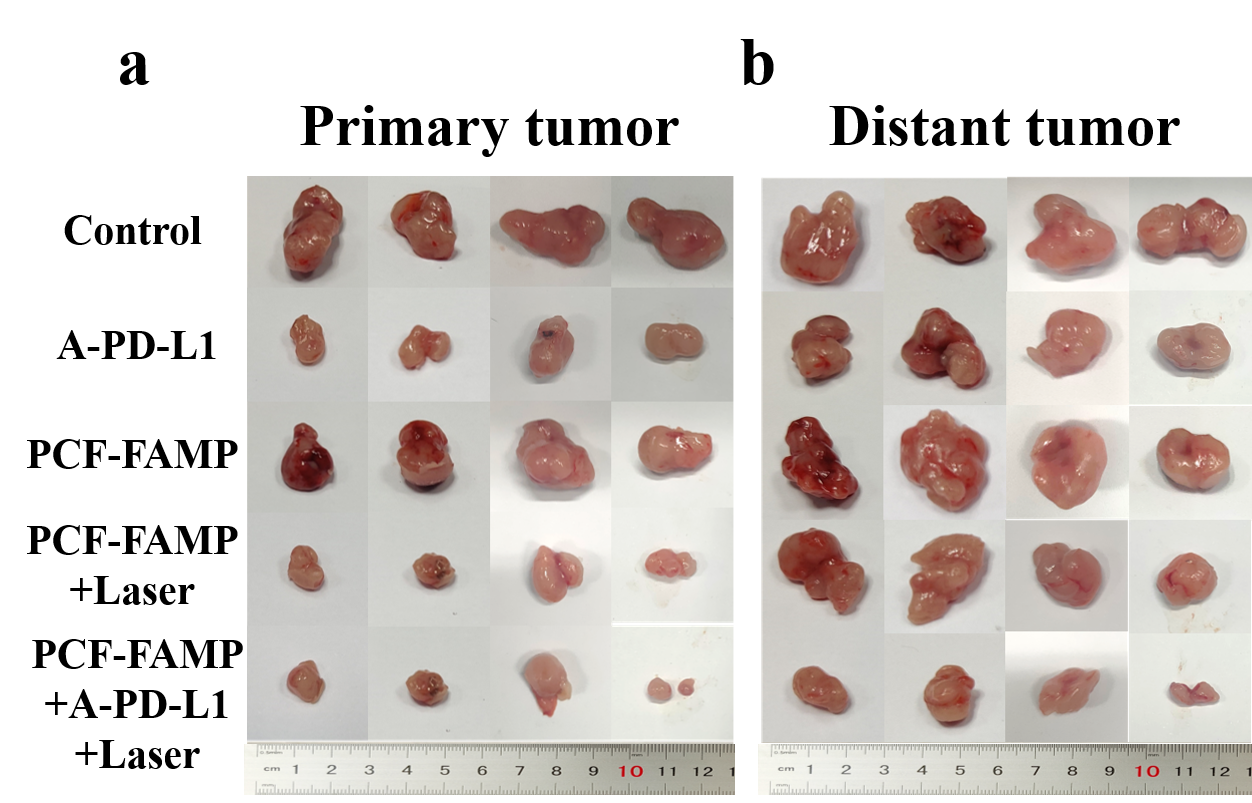


**Figure S10.** (a)-(b) Photographs of tumors dissected from mice of six groups after various treatments.
